# Identification of the ultrahigh-risk subgroup in neuroblastoma cases through DNA methylation analysis and its treatment exploiting cancer metabolism

**DOI:** 10.1038/s41388-022-02489-2

**Published:** 2022-11-01

**Authors:** Kentaro Watanabe, Shunsuke Kimura, Masafumi Seki, Tomoya Isobe, Yasuo Kubota, Masahiro Sekiguchi, Aiko Sato-Otsubo, Mitsuteru Hiwatari, Motohiro Kato, Akira Oka, Katsuyoshi Koh, Yusuke Sato, Hiroko Tanaka, Satoru Miyano, Tomoko Kawai, Kenichiro Hata, Hiroo Ueno, Yasuhito Nannya, Hiromichi Suzuki, Kenichi Yoshida, Yoichi Fujii, Genta Nagae, Hiroyuki Aburatani, Seishi Ogawa, Junko Takita

**Affiliations:** 1 Department of Pediatrics, Graduate School of Medicine, The University of Tokyo, Tokyo, Japan; 2 Department of Pediatrics, Hiroshima University Graduate School of Biomedical Sciences, Hiroshima, Japan; 3 Department of Pediatrics, Teikyo University, School of Medicine, Tokyo, Japan; 4 Department of Hematology/Oncology, Saitama Children’s Medical Center, Saitama, Japan; 5 Department of Urology, The University of Tokyo, Tokyo, Japan; 6 Department of Integrated Analytics, M&D Data Science Center, Tokyo Medical and Dental University, Tokyo, Japan; 7 Department of Maternal-Fetal Biology, National Research Institute for Child Health and Development, Tokyo, Japan; 8 Department of Molecular and Medical Genetics, Gunma University Graduate School of Medicine, Maebashi, Japan; 9 Department of Pediatrics, Graduate School of Medicine, Kyoto University, Kyoto, Japan; 10 Department of Pathology and Tumor Biology, Graduate School of Medicine, Kyoto University, Kyoto, Japan; 11 Division of Brain Tumor Translational Research, National Cancer Center Research Institute, Tokyo, Japan; 12 Genome Science Division, Research Center for Advanced Science and Technology, The University of Tokyo, Tokyo, Japan; 13 Department of Pathology and Tumor Biology, Institute for the Advanced Study of Human Biology (WPI-ASHBi), Kyoto University, Kyoto, Japan

**Keywords:** Paediatric cancer, Cancer metabolism, Targeted therapies, Mechanisms of disease, DNA methylation

## Abstract

Neuroblastomas require novel therapies that are based on the exploitation of their biological mechanism. To address this need, we analyzed the DNA methylation and expression datasets of neuroblastomas, extracted a candidate gene characterizing the aggressive features, and conducted functional studies. Based on the DNA methylation data, we identified a subgroup of neuroblastoma cases with 11q loss of heterozygosity with extremely poor prognosis. *PHGDH*, a serine metabolism-related gene, was extracted as a candidate with strong expression and characteristic methylation in this subgroup as well as in cases with *MYCN* amplification. PHGDH inhibition suppressed neuroblastoma cell proliferation in vitro and in vivo, indicating that the inhibition of serine metabolism by PHGDH inhibitors is a therapeutic alternative for neuroblastoma. Inhibiting the arginine metabolism, which is closely related to serine metabolism using arginine deiminase, had a combination effect both in vitro and in vivo, especially on extracellular arginine-dependent neuroblastoma cells with ASS1 deficiency. Expression and metabolome analyses of post-dose cells confirmed the synergistic effects of treatments targeting serine and arginine indicated that xCT inhibitors that inhibit cystine uptake could be candidates for further combinatorial treatment. Our results highlight the rational therapeutic strategy of targeting serine/arginine metabolism for intractable neuroblastoma.

## Introduction

Neuroblastomas are the second most common malignant solid tumors in children [1–3]. Although some pediatric patients may exhibit spontaneous regression, high-risk patients with metastasis and aged ≥18 months at onset have a long-term recurrence-free survival rate of only approximately 40–50% [4–6]. Furthermore, treatment of high-risk cases requires heavy and long-term combination of chemotherapy, autologous transplantation, tumor resection, and radiation therapy [1]. Hence, short-term complications such as organ damage and infection and long-term complications such as infertility, growth retardation, and hearing loss are significant problems [7].

The heterogeneity in the clinical features suggests heterogeneity in the underlying molecular biological features of each tumor [1]. *MYCN* amplification and loss of heterozygosity (LOH) in chromosome 11q have been reported as prognostic factors [8]. However, even within groups with specific genetic abnormalities, treatment response and disease course varied widely. Therefore, since several years, there has been a demand to elucidate the molecular biological properties of these high-risk neuroblastomas and develop novel therapies based on these properties.

Specific driver genes and corresponding inhibitors have been identified in other malignancies [9, 10]. Accordingly, neuroblastoma has been investigated for genetic mutations using tumor genomic analysis. Through comprehensive copy number analysis of neuroblastoma specimens, Chen et al. identified *ALK* abnormalities in 10% of all neuroblastomas. They observed the involvement of enhanced ALK function in disease pathogenesis [11]. In a Phase-I trial on ALK inhibitors, only 1 of the 11 patients achieved complete remission [12].

However, the approach for single-gene mutations has not been sufficiently practical for identifying therapeutic targets for most patients with neuroblastoma. Pugh et al. performed whole-genome or whole-exon sequencing of 240 neuroblastoma specimens to obtain a comprehensive picture of neuroblastoma mutations [13]. *ALK* mutations accounted for approximately 10%, with six other overlapping mutations accounting for approximately 10%; the remaining 80% did not show overlapping exon mutations. Therefore, most neuroblastoma cases cannot be explained by single mutations and only a small proportion of patients benefit from driver-oriented therapies.

Other approaches to elucidate the disease mechanism and identify targets independent of genetic mutations included expression analysis using RNA sequencing and DNA methylation analysis using DNA methylation arrays and bisulfite sequencing [1, 14, 15]. Westermann et al. reported the results of DNA methylation array analysis of 105 neuroblastomas [16]. They divided the samples into two groups based on their profiles as *MYCN*-amplified and non-*MYCN*-amplified. They isolated the biologically and clinically unique group with *MYCN* amplification, suggesting that DNA methylation profiling can help in biologically meaningful grouping.

Based on this background, we hypothesized that investigating DNA methylation would provide insights into the pathogenesis of neuroblastoma, thus enabling the identification of novel therapeutic targets. In this study, we analyzed DNA methylation in neuroblastoma samples and combined it with RNA sequencing for expression and copy number aberration analyses to clarify the nature of intractable neuroblastomas and extract novel therapeutic targets. We also explored novel therapeutic approaches by focusing on the extracted candidate genes and their related pathways, especially cancer metabolism.

## Results

### DNA methylation analysis defined five clusters in high-risk neuroblastoma

**Fig. 1:**
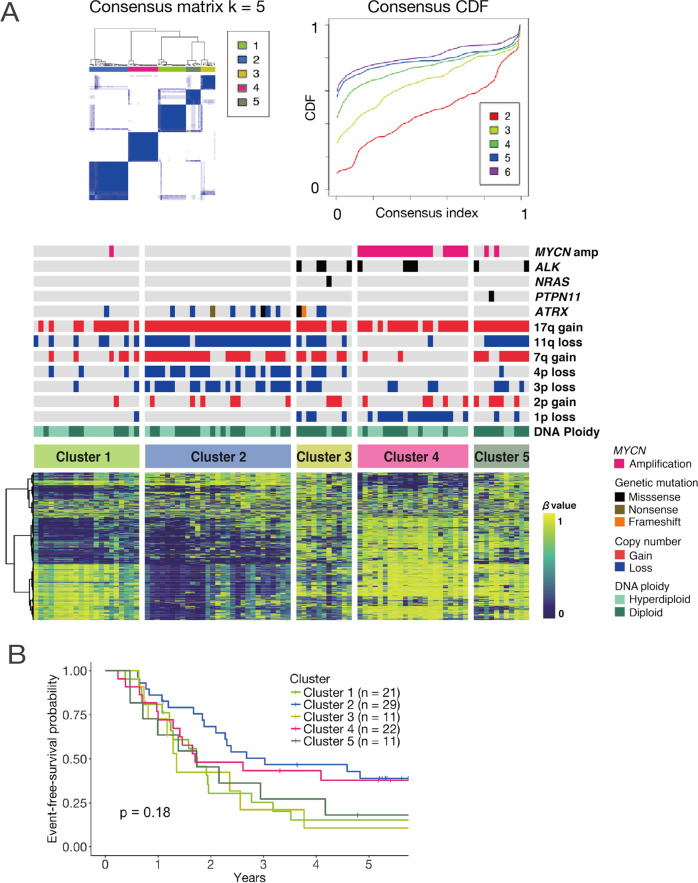
High-risk neuroblastoma cases were classified into five groups based on DNA methylation profiling. **A** Heatmap generated based on the DNA methylation status of 94 high-risk neuroblastomas in the TARGET cohort (bottom). Consensus matrix (upper left) and CDF (upper right) plots indicating that dividing the samples into five clusters was the most suitable approach. **B** Kaplan–Meier plot showing the recurrence-free survival rate for each cluster. There were no significant differences as per the log-rank test.

We first analyzed the DNA methylation status of 94 high-risk neuroblastoma samples in the Therapeutically Applicable Research to Generate Effective Treatments (TARGET) cohort (>1.5 years old at diagnosis and with metastases) [13]. Unsupervised consensus clustering [17] on DNA methylation profiles indicated that it would be appropriate to divide the cohort into five clusters (Fig. 1A). The clinical characteristics of the five groups are presented in Supplementary Table S1. Of the 23 samples showing *MYCN* amplification, 20 were clustered into Cluster 4, which had overall 22 samples. Samples with 11q LOH were mostly clustered into Clusters 2 and 5. *ALK* mutations were found in Clusters 3, 4, and 5. In terms of the age at diagnosis, the cases in Cluster 3 were associated with relatively advanced age than those in other clusters.

Despite the bias among clusters in terms of the proportion of samples with *MYCN* amplification and 11q LOH, there was no significant difference in the event-free survival rate among the five groups as per the log-rank test (Fig. 1B). We also investigated the association between prognosis and the presence or absence of 11q LOH or *MYCN* amplification in the entire cohort and found no significant difference in the event-free survival with or without these factors (Supplementary Fig. S1A, B).

Although our analysis of the entire high-risk neuroblastoma cohort identified a cluster associated with *MYCN* amplifications, no clinically meaningful subgroup with prognostic relevance could be identified. Therefore, we proceeded to conduct a subgroup analysis on samples with established molecular biological features, such as *MYCN* amplification and 11q LOH.

### DNA methylation analysis defined two clusters in neuroblastoma cases with 11q LOH

Regarding the group with *MYCN* amplifications in the TARGET cohort, unsupervised consensus clustering for DNA methylation profiling indicated that classification into three clusters was the most stable option (Supplementary Fig. S1C). However, the number of samples in each cluster was small, and there was no significant difference in the prognosis of these three groups.

**Fig. 2:**
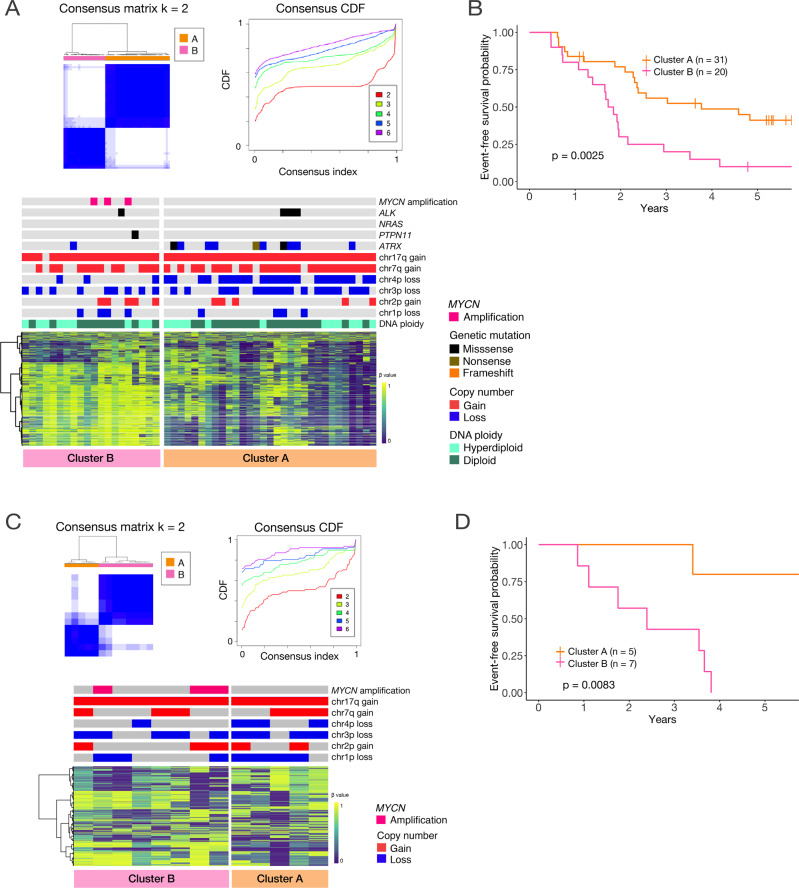
Neuroblastoma cases with 11q LOH can be classified into two groups based on DNA methylation profiling. **A** Results of unsupervised consensus clustering according to DNA methylation profiling for 51 neuroblastomas with 11q LOH in the TARGET cohort. Consensus matrix (upper left) and CDF (upper right) plots indicate that dividing the samples into two clusters was the most suitable approach. Heatmap (bottom) was generated using top 3 000 probes with high variability from the cohort. **B** Kaplan–Meier plot showing the recurrence-free survival rates of each cluster. The prognosis of Cluster B is significantly worse as assessed by the log-rank test. **C**, **D** All 13 cases in the UT cohort were similarly clustered into two groups with different prognoses. Panel **D** presents cases with prognostic information (of a total of 13, wherein one lacked prognostic information).

We further analyzed the DNA methylation status of 51 high-risk neuroblastoma samples with 11q LOH in the TARGET cohort. DNA methylation profiling based on unsupervised consensus clustering identified two clusters with distinct methylation signatures: hypomethylated Cluster A and hypermethylated Cluster B (Fig. 2A). The clinical characteristics of the two groups are presented in Supplementary Table S2. There were no significant differences in terms of sex, INSS stage, and DNA ploidy. Patients in Cluster B had a younger age at diagnosis. Cluster A was characterized by frequent 4p loss and *ATRX* alterations; these alterations were less common in Cluster B. *ALK* mutations were observed in four cases, including three from Cluster A and one from Cluster B. *MYCN* amplification was observed in only three cases in this cohort and were classified into Cluster B. The prognoses of cases in Cluster A were significantly better than cases in Cluster B, which were extremely poor (Fig. 2B). Excluding the three cases with *MYCN* amplification, the results were still statistically significant (Supplementary Fig. S1D).

We used our institutional cohort (UT cohort) to validate these observations. Like the TARGET cohort, we clustered the 11q LOH samples in the UT cohort into two subgroups based on DNA methylation status (Fig. 2C, Supplementary Table S3). A heatmap was generated using the methylation probes with high variability in the UT cohort (Supplementary Table S4). On clustering the UT cohort data using the same probes that were used to cluster the TARGET cohort data (Supplementary Table S5), the samples were further divided into the same two groups with the exception of one sample (Supplementary Fig. S1E). Again, the cluster enriched with 4p loss exhibited significantly better prognosis (Fig. 2D). Therefore, we confirmed that cases with 11q LOH can be classified into two distinct methylation subgroups that correlated well with clinical outcomes.

### PHGDH was extracted as a candidate using integrated analysis

Several previous reports have described the correlation between DNA methylation and gene expression. The hypermethylation of promoter regions causes downregulation of gene expression, whereas hypermethylation in the gene body is often associated with upregulated gene expression [18, 19]. To clarify the molecular mechanisms underlying the aggressive phenotype of Cluster B to identify the therapeutic targets for these intractable neuroblastomas, we analyzed the expression data of the same samples used in DNA methylation analysis using the RNA sequence data of TARGET.

**Fig. 3:**
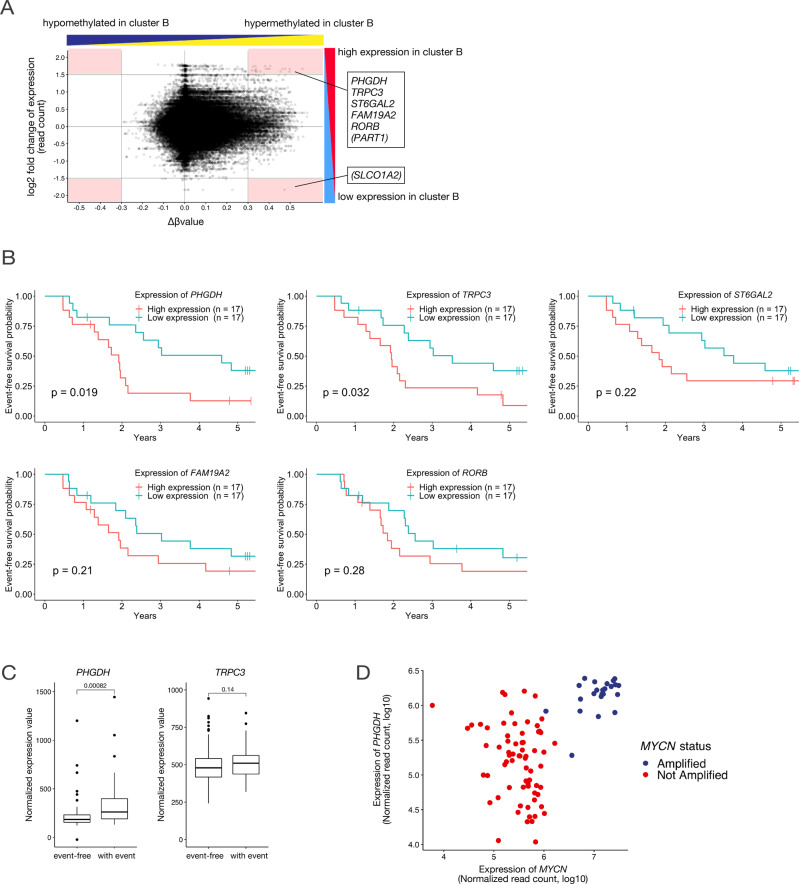
*PHGDH* was extracted as a candidate target gene. **A** Characteristics of the clusters with worse prognosis in terms of DNA methylation status and corresponding gene expressions. Each plot in the starburst plot represents a probe of Infinium Human Methylation 450K BeadChip and its difference in methylation beta values and its fold change in the corresponding gene expression. Positive values indicate higher methylation values or higher expression in Cluster B, which has a worse prognosis in neuroblastomas with 11q LOH. We focused on the plots of probes with differences of ≥0.3 for DNA methylation beta values and ≥1.5 absolute fold changes values of normalized expression counts of corresponding genes (red-colored areas). *PHGDH*, *TRPC3*, *ST6GAL2*, *FAM19A2*, and *RORB* were associated with more than two probes, whereas *PART1* and *SLCO1A2* were associated with only one probe. **B** Kaplan–Meier curves presenting the effect of candidate gene expression on prognosis. Event-free survival rates were compared for cases with the top third expression of each target gene and those with the bottom third expression. *P-*values were determined using log-rank tests. **C** Normalized expression values of *PHGDH* and *TRPC3* in cases with nonamplified *MYCN* and distant metastases at initial diagnosis in the GSE3446 dataset compared with the cases with and without recurrent/progressive events. **D** Relevance of PHGDH expression, *MYCN* expression, and *MYCN* amplification status. The scatterplot represents *PHGDH* and *MYCN* expression in the 94 samples of high-risk neuroblastomas in the TARGET cohort.

The starburst plot presents the differences in the two clusters as identified by DNA methylation profiling in terms of methylation status and corresponding genetic expression (Fig. 3A). To extract those genes that most closely represent the characteristics of Cluster B, we focused on the plots of probes that had absolute differences of ≥0.3 for DNA methylation beta values and absolute fold change values of ≥1.5 for normalized expression counts of corresponding genes. Further, we extracted the genes that revealed an association with more than two of these probes. The top five genes satisfying these conditions were *PHGDH*, *TRPC3*, *ST6GAL2*, *FAM19A2*, and *RORB* (Fig. 3A and Supplementary Fig. S2A). *PART1* and *SLCO1A2* were associated with only one of these probes and were excluded from further analyses. Supplementary Table S6 describes the annotations of these five genes obtained using RefSeq [20]. *PHGDH*, *TRPC3*, *ST6GAL2*, and *RORB* exhibited stronger gene body methylation in Cluster B (Supplementary Fig. S2B). *PHGDH* and *TRPC3* independently predicted worse prognosis based on their expression (Fig. 3B). Furthermore, using another neuroblastoma cohort (GSE3446 [21]), we compared the relationship between the outcomes and *PHGDH* and *TRPC3* expression in cases with metastasis at initial diagnosis without *MYCN* amplification; we found that *PHGDH* expression was stronger in cases with poor outcome but were not different for *TRPC3* (Fig. 3C). In the UT cohort, we observed that gene body hypermethylation was present and *PHGDH* expression was high in Cluster B (Supplementary Fig. S2C, D). Based on these observations, we identified *PHGDH* as a candidate gene whose expression characterizes the aggressive cluster.

*PHGDH* encodes for phosphoglycerate dehydrogenase; this enzyme is essential in the phosphoserine pathway which produces serine in cells [22]. Patients with lung and colon cancers with high *PHGDH* expression have poor prognoses [23, 24]. Enhanced serine biosynthesis by high expression of *PHGDH* has been reported as a metabolic pattern unique to cancer cells (cancer metabolism) [23–25] because it favors tumor cell survival by contributing to DNA and RNA methylation by S-adenosylmethionine [23] and by responding to oxidative stress by glutathione production [22] (Supplementary Fig. S3). Our results indicate the possibility of enhanced serine biosynthesis being a characteristic of clusters with poor prognosis identified in this study.

### High *PHGDH* expression observed in cases with *MYCN* amplification or in the group with 11q LOH and poor prognosis

*MYCN* amplification is an established marker of poor prognosis in neuroblastoma [1, 26]. Previous reports have indicated that the MYCN upregulated *PHGDH* transcription and that *MYCN* amplification status determines the *PHGDH* expression levels [27]. We examined the correlations between *MYCN* status, *MYCN* expression, and *PHGDH* expression in 94 samples with high-risk neuroblastoma, in the TARGET cohort; the expression of the genes in all samples was examined, that is, for cases with and without 11q LOH (Fig. 3D). As previously reported, samples with *MYCN* amplification demonstrated uniformly high *MYCN* and *PHGDH* expression levels, and those without *MYCN* amplification demonstrated low *MYCN* expression levels. However, samples without *MYCN* amplification did not exhibit uniformly low *PHGDH* expression levels. Some cases without *MYCN* amplification exhibited high *PHGDH* expression levels, and these were found to be comparable with cases with *MYCN* amplification.

As described previously, in the TARGET cohort, high *PHGDH* expression is a poor prognostic indicator in cases with 11q LOH independent of *MYCN* amplification. Thus, a high *PHGDH* expression is associated with two important neuroblastoma subgroups: those with *MYCN* amplification and those with 11q LOH and poor prognosis.

To explain the presence of samples with high *PHGDH* expression without *MYCN* amplification, the impact of *MYC* expression or methylation status of the *PHGDH* gene body on *PHGDH* expression was checked as factors of suspected relevance. No correlation between *MYC* expression and *PHGDH* expression was found (Supplementary Fig. S4A), and there were cases where both *MYC* and *MYCN* expression were low, while *PHGDH* expression was high (Supplementary Fig. S4B). Thus, *MYC* expression was unlikely to contribute to the enhanced expression of *PHGDH*. As for the relationship between *PHGDH* gene body methylation and its expression level, the correlation coefficients between the beta value of the probe in the gene body region and its expression level were mostly positive in the group without *MYCN* amplification, with a maximum of 0.68. (Supplementary Fig. S4C, D). Therefore, although the causal relationship was unclear, at least some link between enhanced *PHGDH* expression and enhanced gene body methylation was inferred.

### PHGDH inhibition reduced neuroblastoma cell growth

**Fig. 4:**
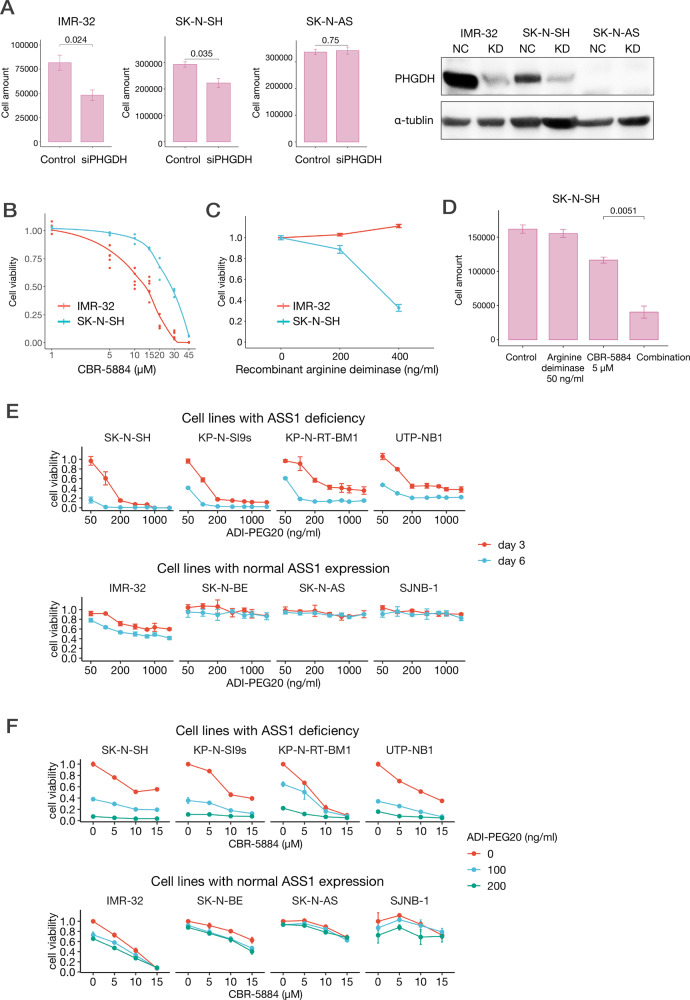
*PHGDH* inhibition and arginine depletion reduce the growth of neuroblastoma cells in vitro. **A** Effect of *PHGDH* knockdown on NB cells. The bar charts (left) indicate the numbers of IMR-32 cells (NB cell line with high *PHGDH* expression without 11q LOH), SK-N-SH cells (NB cell line with relatively low *PHGDH* expression), and SK-N-AS cells (NB cell line that lacks *PHGDH* expression) 48 h after transfection of negative control siRNA or siRNA-targeting *PHGDH*. The bars represent the standard deviations. Western blot analysis images (right). NC, negative control; KD, knockdown. **B** Effect of *PHGDH* inhibitor on NB cell lines. The graph showing the responses of IMR-32 (NB cell line with high *PHGDH* expression) and SK-N-SH (NB cell line with relatively low *PHGDH* expression) to CBR-5884, a PHGDH inhibitor. Cell viability relative to control was evaluated using CellTiter-Glo (Promega). The bars represent standard deviations. **C** Effects of recombinant arginine deiminase on the growth of neuroblastoma cell lines. Cell viability was evaluated using Cell-Counting kit 8. **D** Combination effects of recombinant arginine deiminase and CBR-5884, a PHGDH inhibitor. Combo, combination. **E** Effect of arginine depletion by ADI-PEG20. Cell viability was evaluated using CellTiter-Glo 3 or 6 days after ADI-PEG20 administration. SK-N-SH, KP-N-SI9s, KP-N-RT-BM1, and UTP-NB1 were neuroblastoma cell lines with deficient ASS1. IMR-32, SK-N-BE, SK-N-AS, and SJNB-1 were neuroblastoma cell lines with normal ASS1. **F** Combination effects of ADI-PEG20 and CBR-5884. ADI-PEG20 was administered immediately after seeding and CBR-5884 was administered 24 h after seeding. Cell viability was evaluated using CellTiter-Glo 72 h after the administration of CBR-5884. The combination index was calculated for cell lines with *ASS1* deficiency. The minimal combination indices of SK-N-SH, KP-N-SI9s, KP-N-RT-BM1, UTP-NB1, IMR-32, SK-N-BE, SK-N-AS, and SJNB-1 were 0.78, 0.98, 1.12, 0.55, 0.87, 0.65, 0.66, and 1.44, respectively.

We conducted knockdown assays using siRNA on neuroblastoma cell lines to identify the significance of *PHGDH* expression in aggressive neuroblastoma cells. We used IMR-32, a cell line with high *PHGDH* expression; SK-N-SH, a cell line with relatively low *PHGDH* expression; and SK-N-AS, a cell line without *PHGDH* expression. Inhibition of *PHGDH* by siRNA reduced the growth of IMR-32 and SK-N-SH but not of SK-N-AS (Fig. 4A).

We also conducted an inhibitory study using CBR-5884 (Cayman), a small-molecule compound developed as a *PHGDH* inhibitor [28]. Although CBR-5884 reportedly has no effect on cell lines that lack *PHGDH* expression at concentrations of 30 µM in vitro [28], it reduced the proliferation of neuroblastoma cells in a dose-dependent manner. IMR-32 cells with high *PHGDH* expression exhibited a greater response to CBR-5884 than SK-N-SH cells who had relatively low *PHGDH* expression (Fig. 4B). Since PHGDH was reported to have a protective role against oxidative stress through glutathione production [29], the interaction between N-acetylcysteine (NAC), a radical scavenger, and PHGDH inhibition in neuroblastoma was examined. NAC partially reduced the cytotoxicity of PHGDH inhibitors on neuroblastoma cell lines in vitro (Supplementary Fig. S5), indicating that PHGDH works for oxidative stress reduction in neuroblastoma.

These observations suggest that PHGDH can be a novel therapeutic target for neuroblastoma. As *PHGDH* expression is associated with *MYCN* amplification and poor prognosis with 11q LOH, PHGDH inhibitors would be useful in these aggressive cases.

### Arginine metabolism is closely associated with *PHGDH*

Metabolism in tumor cells involves complex networks; several genes, enzymes, and metabolites affect each other and enable proliferation and growth [30]. Enhanced serine production by PHGDH helps tumor growth and is considered as a pattern of cancer metabolism [24]. Another example of cancer metabolism is the abandonment of arginine synthesis due to ASS1 deficiency [31]. ASS1-deficient tumor cells can spare more cellular aspartate for the biosynthesis of nucleotides and proteins and are dependent on extracellular arginine. Arginine depletion by arginine deiminase reduces the growth of several types of ASS1-deficient tumors [32]. Another recent report by Kremer et al. [33] also stated that arginine depletion in a particular type of ASS1-deficient sarcoma induced the enhancement of serine-glycine biosynthetic pathway through enhanced *PHGDH* expression and attenuated Warburg effect. These changes were reported to lead to dependence on serine metabolism mediated by PHGDH and enhanced the effect of PHGDH inhibitors.

To investigate the combinational treatment of PHGDH inhibition and arginine depletion in neuroblastomas, we confirmed *ASS1* expression levels in neuroblastomas and their correlation with *PHGDH* expression levels. We found no clear correlation between their expression intensities in the TARGET cohort (correlation coefficient = 0.18). With respect to cases with weak *PHGDH* expression, the expression intensity of *ASS1* was also found to be relatively weak; however, this trend, although widely observed, was not uniform across the cohort (Supplementary Fig. S6A). Although PHGDH inhibitors are unlikely to exert strong effects in cells with weak *PHGDH* expression, we hypothesized that arginine depletion alone or in combination with PHGDH inhibitors has a therapeutic effect in cells with weak *ASS1* expression, which is also found in some of the cells with weak *PHGDH* expression.

### There is a neuroblastoma group with no *ASS1* expression

To confirm that there were cases of *ASS1* deficiency in neuroblastoma, its expression in eight neuroblastoma cell lines was first observed by RT-PCR and Western blotting. *ASS1* expression was detected in IMR-32, SJNB-1, SK-N-BE, and SK-N-AS, but not in SK-N-SH, KP-N-SI9s, KP-N-RT-BM1, and UTP-NB1 (Supplementary Fig. S6B, C). Given that UTP-NB1 is a cell line established from a clinical sample in our institute, the expression of *ASS1* in the cell line and samples from the UT cohort was examined. Based on RNA sequencing and RT-PCR results, about half of the samples, including the clinical samples from which UTP-NB1 was established, were deficient in *ASS1* expression (Supplementary Fig. S7A, B). There were no significant differences in prognosis based on *ASS1* expression status (Supplementary Fig. S7C).

### Arginine depletion reduces the growth of ASS1-deficient neuroblastoma cells and shows synergistic effects with PHGDH inhibition

To examine the effects of arginine depletion in neuroblastomas, we investigated the effects of arginine deiminase in cell lines in vitro. To simulate arginine depletion, we added recombinant arginine deiminase (Peprotech) or pegylated arginine deiminase (ADI-PEG20) to the culture medium.

Both recombinant arginine deiminase and ADI-PEG20 reduced *ASS1-*deficient neuroblastoma cell proliferation in a dose-dependent manner (Fig. 4C, E). However, ADI-PEG20 exhibited greater effects at low doses than recombinant arginine deiminase (Fig. 4E). In contrast, with normal *ASS1*-expressing neuroblastoma cells exhibited only mild to no response to arginine depletion.

We also investigated the combination effect of arginine depletion and PHGDH inhibition in vitro. We found that a combination of recombinant arginine deiminase or ADI-PEG20 and CBR-5884 synergistically reduced the viability of *ASS1-*deficient neuroblastoma cells (Fig. 4D, F). The combination index [34] of these two therapies was 0.78 in SK-N-SH and 0.55 in UTP-NB1.

### PHGDH inhibitor and arginine deiminase reduced neuroblastoma cell growth in vivo

**Fig. 5:**
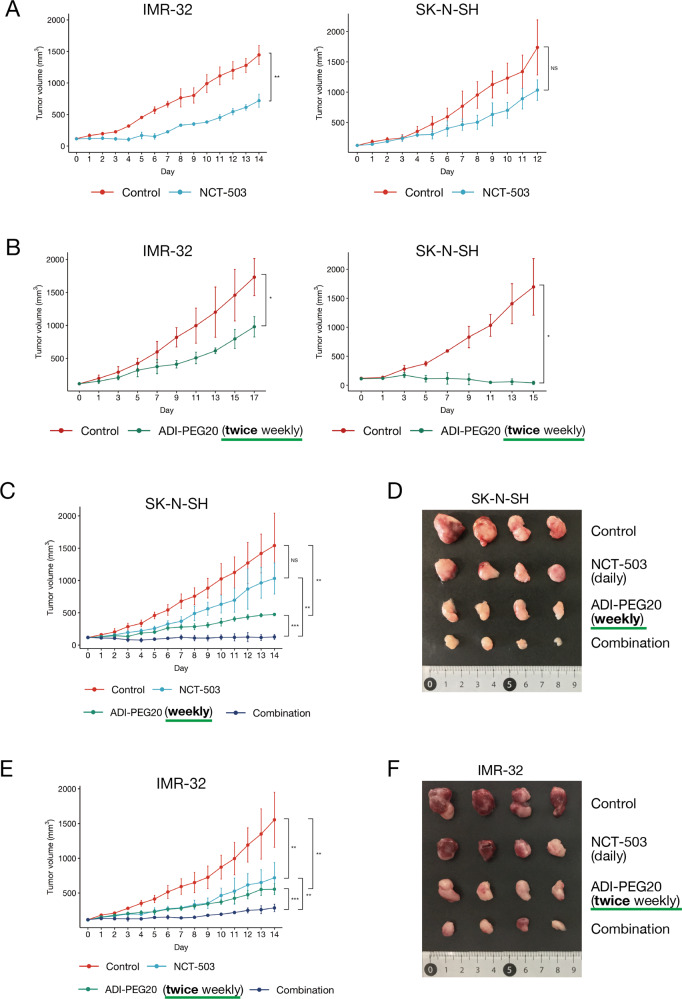
Effects of *PHGDH* inhibitors, ADI-PEG20, and their combination in a tumor mouse model. Each experiment was performed in quintuplicates (five tumors on five mice per condition). All treatments were initiated on the day the tumor size reached 100 mm^3^. The bars represent standard deviations. **A** Effect of NCT-503, a *PHGDH* inhibitor, on a neuroblastoma xenograft mouse model. NCT-503 (40 mg/kg) was administered daily. **B** Effect of ADI-PEG20 on a mouse xenograft model. ADI-PEG20 was administered twice a week. **C–F** Combination effect of NCT-503 and ADI-PEG20. NCT-503 was administered daily. For IMR-32 xenograft mice, ADI-PEG20 was administered twice a week. For SK-N-SH xenograft mice, ADI-PEG20 was administered once a week. After 14 days from the initial dose, the mice were sacrificed. Excised tumors are shown in Figs. 5D and 5F. Only four tumors for each condition were shown due to the gaps in the date of excisions.

To confirm the abovementioned results in vivo, we administered PHGDH inhibitor and ADI-PEG20 to the NOG/SCID neuroblastoma mice model. We first examined the effect of a single agent; for this, we used NCT-503 [35] as a PHGDH inhibitor in vivo because of its better stability in vivo. NCT-503 (40 mg/kg, daily) significantly reduced the growth of IMR-32 cells grafted on NOG/SCID mice. In contrast, the effect against SK-N-SH cells was relatively mild with no significant difference (Fig. 5A). ADI-PEG20 administration (15 mg/kg) twice a week almost completely stopped the growth of SK-N-SH cells in vivo and retarded the growth of IMR-32 cells (Fig. 5B). We further investigated the combination effects of these two agents (Supplementary Fig. S8A). In the combinational study, we reduced the administration of ADI-PEG20 to once a week only in SK-N-SH cells. There was a moderate but significant inhibition of growth even after reducing ADI-PEG20 administration to once a week in SK-N-SH cells. The combination of daily NCT-503 and weekly ADI-PEG20 administration almost halted tumor growth (Fig. 5C, D). We also evaluated the effects of ADI-PEG20 administered twice a week and NCT-503 administered daily on IMR-32 cells and observed significant combination effects (Fig. 5E, F). There were no adverse effects for either of the drugs in terms of body weight or other findings (Supplementary Fig. S8B).

### Arginine depletion altered cancer metabolism to support PHGDH inhibition

**Fig. 6:**
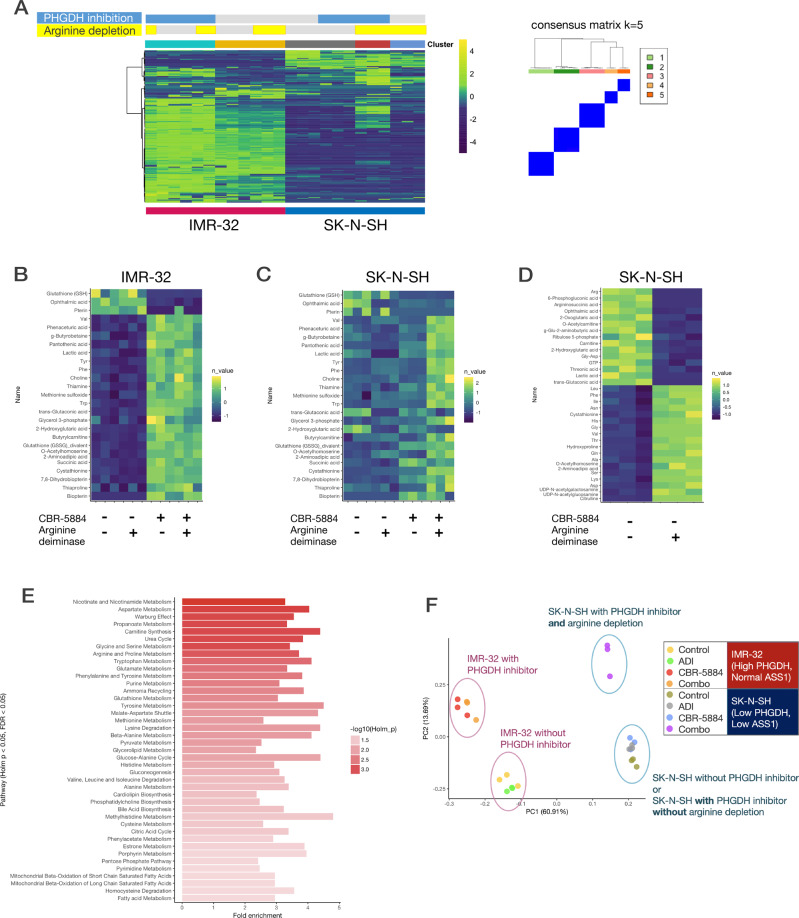
Metabolome analysis showing that arginine deiminase altered cancer metabolism to enhance the effect of PHGDH inhibitor. **A** Heatmap (left) shows the relative amounts of metabolites extracted from neuroblastoma cells treated with or without CBR-5884 and arginine deiminase. There were four conditions (with or without PHGDH inhibitor and with or without arginine deiminase) on two neuroblastoma cell lines. Experiments with each sample were performed in triplicates for each condition. Consensus plot (right) indicating that the five stable clusters in the 24 samples were defined via unsupervised consensus clustering. **B** Relative amounts of top affected metabolites whose levels were most affected by CBR-5884 (PHGDH inhibitor) in IMR-32 cells. **C** Relative amounts of the same metabolites described in Fig. 6B in SK-N-SH cells. **D** Relative amounts of top affected metabolites whose levels were affected by arginine deiminase treatment in SK-N-SH cells. **E** Pathways affected by arginine deiminase in SK-N-SH cells. The bar chart shows the magnitude of changes in the pathways extracted using metabolite set enrichment analysis. **F** Principal component analysis plot of metabolite profiling. ADI arginine deiminase, Combo combination of PHGDH inhibitor and arginine deiminase.

We conducted metabolome analysis using post-dose cell lines to better elucidate the therapeutic effects of PHGDH inhibition and arginine depletion (Supplementary Fig. S9). For this purpose, we performed capillary electrophoresis and time-of-flight mass spectrometry [36] using two neuroblastoma cell lines with or without CBR-5884 and arginine deiminase and compared the treatment effect on IMR-32 (high *PHGDH* expression, normal ASS1) and SK-N-SH (low *PHGDH* expression, deficient ASS1) cells. There were four conditions for two cell lines and all experiments were conducted in triplicate, resulting in 24 samples in total. Supplementary Table S7 presents the detected metabolites. Supplementary Fig. S10 maps the relative levels of the detected metabolites. Unsupervised consensus clustering of the obtained metabolite profiles of 24 samples indicated five stable clusters (Fig. 6A, right). As anticipated, IMR-32 cells exhibited changes that were primarily induced by the PHGDH inhibitor (Fig. 6A, left, and 6B); it reduced the levels of glutathione and ophthalmic acid, both of which are downstream products of serine metabolism. However, it did not reduce the serine or glycine levels. Conversely, it increased the amount of lactate, a downstream product of the glycolytic system. In contrast, the metabolite profiles of SK-N-SH cells were primarily altered by arginine depletion, whereas combination therapy exerted different effects (Fig. 6A, right, C, D). Arginine depletion in SK-N-SH increased the amount of serine and glycine and decreased the amount of lactate. The major changes observed with the administration of CBR-5884 in IMR-32 cells were seen to appear in SK-N-SH cells with the combination therapy (Fig. 6B, C).

Metabolite set enrichment analysis [37] further extracted the intracellular metabolic pathways that were altered by each treatment for each cell line. PHGDH inhibition on IMR-32 altered several metabolic pathways, but no significant changes in glycine and serine metabolism were detected (Supplementary Fig. S11A) since their amounts were unchanged. Of note, Warburg effect was significantly enhanced, suggesting that PHGDH inhibition shifted glucose metabolism from glucose-derived serine biosynthesis to an anaerobic metabolism pathway (Supplementary Fig. S11B). In contrast, arginine deiminase upregulated the glycine and serine metabolism and downregulated the Warburg effect on SK-N-SH cells (Fig. 6E and Supplementary Fig. S11C).

We also performed principal component analysis of the metabolite profiling (Fig. 6F). PC1 primarily corresponded to the differences in cells. For IMR-32 cells, PC2 primarily corresponded to the effect of PHGDH inhibition. For SK-N-SH cells, changes in PC2 were observed only in the combination therapy group. The factors indicated that PC2 was most prominent with the increase of glycerol 3-phosphate, the metabolite located upstream of 3-phosphoglycerate (3PG) where PHGDH acts in the glycolytic pathway, and the decrease of glutathione, downstream of the serine production pathway. This observation suggested that adding arginine deiminase enhanced the effect of PHGDH inhibition in the metabolite profiling of cells with relatively low *PHGDH* expression and deficient *ASS1*.

### PHGDH inhibition and arginine depletion enhanced the compensatory uptake of cystine into neuroblastoma cells

**Fig. 7:**
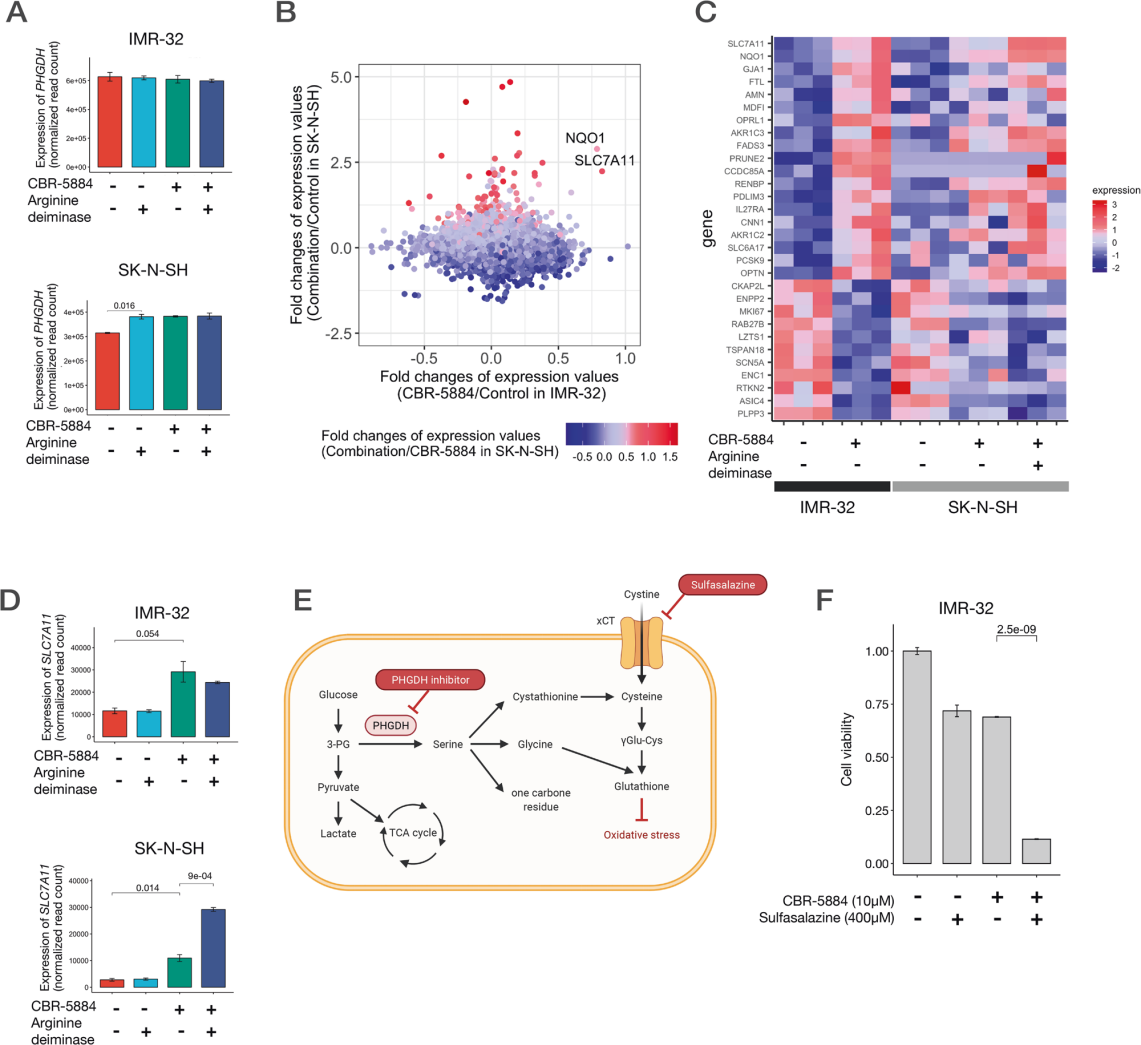
Inhibition of the serine biosynthetic pathway enhances the expression of *SLC7A11*, and its coinhibition results in additional therapeutic effects. **A** Changes in *PHGDH* expression in cell lines by CBR-5884 and arginine deiminase. Expression values were normalized read counts obtained from the RNA sequence. IMR-32 cells did not show any significant changes in *PHGDH* expression, whereas SK-N-SH cells showed enhanced *PHGDH* expression after treatment with arginine deiminase or CBR-5884. **B** Changes in gene expressions of neuroblastoma cell lines induced by CBR-5884 and arginine deiminase. The effects of CBR-5884 in IMR-32 cells are presented on the X-axis and those of a combination of CBR-5884 and arginine deiminase in SK-N-SH cells are indicated on the Y-axis. The effects of addition of arginine deiminase to CBR-5884 in SK-N-SH cells are indicated by color. **C** Heatmap of the expression levels of genes whose expressions were altered by CBR-5884 treatment in IMR-32 cells and altered in the same direction by the combination of two drugs in SK-N-SH cells. The genes were arranged by the magnitude of change in expression levels by CBR-5884 in IMR-32 cells. **D** Changes in *SLC7A11* expression by the two drugs; enhanced in IMR-32 cells by CBR-5884 and enhanced significantly in SK-N-SH cells by the combination of the two drugs. **E** Schema illustrating the relationship of the metabolic pathways associated with serine, glycine, cystine, PHGDH, and *SLC7A11*. Both gene products are associated with glutathione production. **F** Combination effect of CBR-5884 and sulfasalazine on IMR-32 cells. The drugs were administered 24 h after seeding. Cell viability was found to be evaluated using CellTiter-Glo 72 h after the administration of the drugs. Panel **E** was created using BioRender.com.

To determine the cellular responses with PHGDH inhibition and arginine depletion in neuroblastoma cells, we conducted transcriptome analysis using post-dose cell lines under the same condition as those in the metabolome analysis (Supplementary Fig. S9). We extracted RNA from the treated cells and sequenced it to observe the changes in gene expression induced by the treatments. In SK-N-SH cells, *PHGDH* expression was upregulated not only by the PHGDH inhibitor but also by arginine deiminase (Fig. 7A), which is concordant with the hypothesis that arginine depletion induces dependence on serine metabolism. We extracted genes that most represent the combination effects of PHGDH inhibition and arginine depletion, i.e., genes upregulated by PHGDH inhibition in IMR-32 cells, by the combinational therapy in SK-N-SH cells, and by the combinational therapy in comparison with PHGDH inhibition alone in SK-N-SH cells (Fig. 7B–D). The top two representative genes were *SLC7A11* and *NQO1*. *SLC7A11* encodes the xCT channel protein responsible for cystine uptake into cells and is reported to be upregulated in some tumor cells to produce glutathione in response to oxidative stress [38]. *NQO1* encodes an enzyme with antioxidant properties and responds to oxidative stress in tumor cells [39, 40]. As serine metabolism is essential for glutathione production and PHGDH inhibitor reduces glutathione production, upregulating the expression of *SLC7A11* and *NQO1* should compensate for the glutathione exhausted by these treatments (Fig. 7E).

### Inhibition of xCT is a candidate approach for combination therapy with PHGDH inhibition

By combining the results of metabolome and expression analyses, we hypothesized that xCT inhibition enhances the effect of PHGDH inhibition in a manner similar to synthetic lethality. We conducted a compound assay using sulfasalazine, an xCT inhibitor, with a combination of PHGDH inhibitors. As expected, sulfasalazine demonstrated synergetic effects with PHGDH inhibitor (Fig. 7F). Therefore, xCT inhibition would be a good candidate for combination therapy.

## Discussion

In a previous study on DNA methylation in neuroblastomas, methylation profiling reportedly distinguished samples with *MYCN* amplification [16]. However, because *MYCN* is a known transcription factor and a prognosis factor with established detection methods [1], the unique significance of DNA methylation analysis has been investigated. We succeeded in isolating the ultrahigh-risk group among cases with 11q LOH via DNA methylation analyses in two cohorts, suggesting that methylation analysis can identify groups with distinct biology and prognosis that is not possible using other methods. Hence, DNA methylation profiling has potential usage as a risk-stratification and risk-adapted therapy for neuroblastoma.

Based on the results of combined data analyses, we extracted *PHGDH*, a gene encoding the enzyme essential in serine biosynthesis [24], as a potential target. To elucidate the characteristics of poor prognosis clusters of neuroblastoma with 11q LOH, genes exhibiting strong characteristics in terms of DNA methylation and gene expression were selected. The cluster with poor prognosis was mostly characterized by the enhanced methylation of the gene body region of *PHGDH* and its enhanced expression. It was reported that the hypermethylation of the gene body region is likely to be associated with a high gene expression [19], but the causal relationship is often unclear. Moreover, the cluster with poor prognosis showed an overall trend of hypermethylation. While the methylation status of the *PHGDH* gene body might affect its expression, it is also possible that a strong *PHGDH* expression may contribute to its extensive hypermethylation in the cluster with poor prognosis by enhancing the serine biosynthetic pathway, causing an increased S-adenosylmethionine production, which is its downstream product that mediates DNA methylation. It is difficult to determine the cause and result.

In terms of the relationship between *MYCN* status and *PHGDH* expression in neuroblastoma, Xia et al. reported that neuroblastoma cells with *MYCN* amplification showed high *PHGDH* expression and were sensitive to PHGDH inhibitors [27]. They also reported that cases without *MYCN* amplification had low *PHGDH* expression and that inhibitory drugs were effective for cases with *MYCN* amplification. However, we demonstrated that cases without *MYCN* amplification showed varying levels of *PHGDH* expression. Some cases without *MYCN* amplification, with relatively low expression of *MYCN*, exhibited strong *PHGDH* expressions similar to those with *MYCN* amplification. In cases with 11q LOH, which rarely had *MYCN* amplification, high *PHGDH* expression was a poor prognostic factor regardless of the *MYCN* amplification status. Therefore, *PHGDH* expression is also important in neuroblastoma without *MYCN* amplification as a prognostic factor as well as a therapeutic target.

Serine biosynthesis pathway mediated by PHGDH has been recognized as one of the patterns of cancer-specific metabolism, and its inhibition has been reported to have specific therapeutic effects on several cancer cells with high *PHGDH* expression. Therefore, inhibitors of PHGDH, such as CBR-5884 and NCT-503, have been developed [28, 35]. The doses of CBR-5884 and NCT-503 used on neuroblastoma cell lines were similar to or lower than those reported to be effective in other cancer cell lines in prior reports [28, 35]. Regarding the PHGDH inhibitor against neuroblastoma, Arlt et al. reported its effect on PDX models of neuroblastoma [41]. They reported that NCT-503 slowed proliferation of neuroblastoma in vivo but had a limited effect in killing cells; they concluded that PHGDH inhibition has limited potential as a treatment option for neuroblastoma. Our results also showed that PHGDH inhibitor alone slowed but did not completely stop the growth of tumors in vivo. Therefore, combination with other drugs is required to achieve enough therapeutic effect.

*ASS1* expression, which is required for intracellular arginine production, is reportedly weak in some malignancies [32]. These *ASS1*-deficient cells depend on arginine uptake from extracellular sources [31] and are susceptible to extracellular arginine depletion; thus, potential treatment by arginine depletion has been proposed [42, 43]. Regarding neuroblastoma, Fultang et al. reported that arginine depletion with recombinant arginase (BCT-100) prolonged the survival of TH-MYCN mice by some days [44]. We demonstrated that ADI-PEG20, a pegylated arginine deiminase, almost halted the growth of neuroblastoma cells with ASS1 deficiency and reduced the growth of those without ASS1 deficiency for 2 weeks in vivo. The strong effects observed in ADI-PEG20 may be attributable to the differences in the point of action and stability of the drugs due to pegylation.

Some *ASS1*-deficient sarcomas reportedly exhibit compensatory upregulated serine synthesis and increased sensitivity to PHGDH inhibitors in response to arginine depletion [33]. *PHGDH* expression was enhanced in neuroblastoma cells with ASS1 deficiency treated with arginine deiminase, with a concomitant enrichment of intracellular serine and glycine and downregulation of the Warburg effect. Our findings demonstrated the excellent combination effects of PHGDH inhibitor and ADI-PEG20 for neuroblastoma in vivo. The combinational therapy was also effective for IMR-32 cells, i.e., neuroblastoma cells without *ASS1* deficiency, particularly in vivo. This intense effect in vivo could be because the amino acids obtained in vivo were limited compared with those obtained from culture medium in vitro. Combining these two drugs could be potentially effective for a wide variety of neuroblastomas, irrespective of the presence or absence of *ASS1* deficiency.

Metabolome analysis allows a complete view of the pathways through which the intervention alters intracellular metabolic dynamics [36]. Using metabolome set enrichment analysis [37], we confirmed that PHGDH inhibitors attenuates its downstream metabolism and that arginine depletion enhances serine and glycine metabolism as a compensatory change. Moreover, we confirmed that the metabolic effects of PHGDH inhibitors in cells with strong *PHGDH* expression were observed only under conditions of arginine depletion in cells with relatively weak *PHGDH* and deficient *ASS1* expressions, thus confirming their combination effects.

Although PHGDH was reported to be essential for intracellular serine biosynthetic pathway [45], the administration of PHGDH inhibitors did not decrease the serine or glycine levels in neuroblastoma cells. Based on initial reports on the importance of PHGDH in cancer cells, PHGDH inhibition does not decrease intracellular serine levels [35, 45]. In these reports, tracing methods using 13C and other tracers showed that the amount of intracellular serine is maintained by uptake of extracellular serine and synthesis from glycine, while its downstream product, glutathione, is decreased. Therefore, the effects of PHGDH inhibition cannot be verified by measuring serine alone. A limitation of this study was that the origin of intracellular serine was not verified using tracing methods; moreover, we cannot completely rule out the possibility that the effect of PHGDH inhibitors is due to off-target effects. However, metabolomic analysis showed that PHGDH inhibition enhanced the anaerobic glycolysis and decreased the glutathione levels. These results indicate that the flow of glucose metabolism was switched from the direction of serine biosynthesis pathway to the downstream direction of glycolytic system, indicating that PHGDH inhibitors induce metabolic changes in neuroblastoma similar to those previously reported for other malignancies. The attenuated effects of PHGDH inhibitors by NAC also demonstrate that PHGDH contributes to oxidative stress reduction in neuroblastoma through glutathione production and that PHGDH inhibitors block this function.

We observed that PHGDH inhibition enhanced the expression of *SLC7A11* (xCT) and that sulfasalazine, which reportedly inhibits xCT [46], is a potential candidate for combination therapy. Sulfasalazine was originally used as an antirheumatic drug; however, studies have reported its therapeutic effects on leukemia cells through xCT inhibition [46], making it an attractive candidate drug for various tumors to reduce cells via the inhibition of glutathione production and loss of resistance to oxidative stress [47, 48]. In this study, we proposed that the therapeutic effect of PHGDH inhibitors could be enhanced by simultaneously inhibiting xCT and interfering in the production of glutathione. As stated before, combining drugs to interfere with the multiple tumor metabolisms is expected to have a stronger therapeutic effect. While the metabolome and expression analyses were performed in vitro, similar analyses of tumor tissue and plasma using a tumor mice model would provide a more accurate evaluation of the effects of drugs in vivo and optimization of combination therapy. We are currently conducting these studies.

We proposed a candidate treatment that is expected to be particularly effective for neuroblastomas with enhanced PHGDH expression, i.e., with *MYCN* amplifications and 11q LOH and poor prognosis. As established targeted therapies were lacking for such cases [1, 6], it would be helpful to develop a novel treatment for these cases. Furthermore, as cancer metabolism is a characteristic of certain cancer cells, therapies targeting these metabolic patterns are expected to have less effect on normal cells [30], as was observed in our in vivo study. Therefore, these therapies may show fewer adverse events than conventional chemotherapy and can be used in combination with existing therapies. In particular, attenuating glutathione production by PHGDH inhibitors may enhance the effect of various chemotherapies by attenuating the resistance of neuroblastoma cells to oxidative stress.

In conclusion, our multiomic study revealed that DNA methylation profiling could help in identifying subgroups with worse prognoses among high-risk neuroblastoma cases. Moreover, simultaneous inhibition of serine and arginine metabolism is a candidate approach for combination therapy for neuroblastomas. Further clinical investigations are warranted for practical use.

## Materials and methods

Details of all of the methods are provided in the online supplement.

## Supplementary information

Supplementary materials and methods

Supplementary Figure S1

Supplementary Figure S2

Supplementary Figure S3

Supplementary Figure S4

Supplementary Figure S5

Supplementary Figure S6

Supplementary Figure S7

Supplementary Figure S8

Supplementary Figure S9

Supplementary Figure S10

Supplementary Figure S11

Supplementary Table S1

Supplementary Table S2

Supplementary Table S3

Supplementary Table S4

Supplementary Table S5

Supplementary Table S6

Supplementary Table S7

Supplementary Table S8

Supplementary Table S9
