# Supplementary material for: Identification of the ultrahigh-risk subgroup in neuroblastoma cases through DNA methylation analysis and its treatment exploiting cancer metabolism
Source: Oncogene. 2022 Nov 1;41(46):4994–5007. doi: 10.1038/s41388-022-02489-2 (PMC9652143; doi:10.1038/s41388-022-02489-2)
Supplement: Supplementary file 1 — Supplementary materials and methods [file 41388_2022_2489_MOESM1_ESM.docx]

Supplementary materials and methods

Samples in our institutional cohort

Our institutional cohort (UT cohort) comprised 28 tumors from high-risk neuroblastoma cases diagnosed based on biopsy findings. High-risk cases were defined as those aged >1.5 years at the time of diagnosis and with metastases. We conducted targeted capture sequencing and calculated the copy number as previously reported [1] and extracted 13 samples with 11q LOH (DNA Data Bank of Japan [DDBJ] accession number hum0035, JGAS000246). Supplementary Table S8 provides detailed information on the participants and samples. The patients’ parents provided written informed consent according to protocols approved by the Human Genome, Gene Analysis Research Ethics Committee of the University of Tokyo (approval number G1598).

Obtaining public datasets

We downloaded the datasets generated by the Therapeutically Applicable Research to Generate Effective Treatments (TARGET, https://ocg.cancer.gov/programs/target) initiative phs000467. The datasets included the data of DNA methylation analysis performed using the Infinium HumanMethylation450 BeadChip (Illumina) and RNA sequencing performed using Hiseq2000 (Illumina). Copy number alteration data were obtained from a previous study [2]. We also obtained the datasets of expression arrays deposited under GSE3446 [3] in the Gene Expression Omnibus (GEO, RRID:SCR_005012, <http://www.ncbi.nlm.nih.gov/geo/>) [4].

DNA methylation analysis

We performed comprehensive DNA methylation analysis on the 13 samples from the UT cohort using the Infinium HumanMethylation450 BeadChip (Illumina) as per the manufacturer’s protocol.

To analyze the DNA methylation data of the UT and TARGET cohorts, we first used the ChAMP package (RRID:SCR_012891) in R to obtain normalized beta values from the IDAT files for each cohort [5]. We then eliminated the SNP-related probes, non-CpG probes, and probes designed for sequences on the X and Y chromosomes. We only selected those probes with variance that ranked top 1000–5000 from the remaining probes (Supplementary Tables S4, S5, and S9). With the matrix data of selected beta values, we conducted unsupervised consensus clustering using the ConsensusClusterPlus package (RRID:SCR_016954) [6] to identify the most stable clusters.

RNA sequencing and gene expression analysis

For the UT cohort for which RNA was available, RNA sequencing libraries were prepared using the NEBNext Ultra RNA Library Prep kit from Illumina (New England BioLabs). Next-generation sequencing was performed using the Illumina HiSeq 2000 or 2500 platform with a standard 100-bp paired-end read protocol according to the manufacturer’s instructions [1].

We first performed alignment and quality checks to analyze the RNA sequencing data for the UT and TARGET cohorts and obtained the read count data using our pipeline known as Genomon (<http://genomon.hgc.jp/exome/en/index.html>) for each dataset. The obtained read count data were normalized using the variance-stabilizing transformation of the R package DESeq2 (RRID:SCR_000154) [7]. Differential expression analysis was also performed using the Wald test with negative binomial generalized linear model fitting.

Cell lines and cell culture

All cell lines were confirmed to be mycoplasma-free based on VenorGeM OneStep (Minerva Biolabs). The RIKEN Cell Bank provided the IMR-32 cell lines; Dr. Takagi of Tokyo Medical and Dental University provided the SK-N-SH and SK-N-BE cell lines; and Dr. Umeda of Kyoto University provided the SK-N-AS cell lines. The Japanese Collection of Research Bioresources Cell Bank provided the KP-N-SI9s and KP-N-RT-BM1 cell lines. Dr. T.A. Look of St. Jude Children’s Research Hospital provided the SJNB-1 cell lines. UTP-NB1 was established from a patient’s tumor tissue at the University of Tokyo Hospital. These cell lines were grown in RPMI 1640 Media (Thermo Fisher Scientific), supplemented with heat-inactivated 10% fetal bovine serum (Gibco) and 100 U/ml penicillin–streptomycin (Gibco) at 37°C in a 5% CO_2_ humidified incubator.

siRNA transfection

PHGDH knockdown was performed on the neuroblastoma cell lines. We seeded cells in 24-well plates (Corning) at 30,000–50,000 cells/well and transfection was performed 24 h after seeding. We transfected two siPHGDH (Silencer Select siRNA) and one negative control siRNA (Silencer Select Negative Control siRNA No.1) using Lipofectamine RNAiMAX (Thermo Fisher Scientific) [8]. We counted the cells using trypan blue solution (Sigma) after incubating for 72 h.

Western blot analysis

Protein lysates were prepared using the RIPA lysis buffer and separated on 4%–12% Mini-PROTEAN TGX Precast Gels (BIO-RAD), transferred onto a nitrocellulose membrane (Millipore), and incubated with α-tubulin (Abcam, catalog ab7291), PHGDH (CST, catalog 66350) [9] , and ASS1 (CST, catalog 70720) antibodies.

Cell proliferation assays

Cells were seeded in 96-well plates (Corning) at a concentration of 2000–3000 cells/well. Polaris Pharmaceuticals, Inc. (San Diego, CA, USA) provided ADI-PEG20 [10]. We added recombinant arginine deiminase (Abcam) and ADI-PEG20 to the medium immediately after seeding. CBR-5884 (PHGDH inhibitor, [11] Cayman), N-acetylcysteine (Sigma, catalog B4894), and sulfasalazine (Cayman) were added to the medium 24 h after seeding. Cell viability was evaluated using CellTiter-Glo2 (Promega).

Metabolome analysis

Tumor cells were seeded in 10-cm dishes (Corning) at 4 × 10^5^ cells/dish (IMR-32) or 6 × 10^5^ cells/dish (SK-N-SH) in the culture medium, with or without recombinant arginine deiminase (Abcam), at a final concentration of 200 ng/ml. After 96 h, we added CBR-5884 (Cayman) at a final concentration of 10 μM or the corresponding dose of DMSO as a negative control to the medium. We conducted all experiments in triplicates. After another 24 h, we extracted intracellular metabolites and conducted metabolome measurements through a facility service at Human Metabolome Technologies Inc., Tsuruoka, Japan.

The obtained concentrations of the metabolites were normalized by cell counts. Detected metabolites were plotted on the metabolic pathway maps using the VANTED software [12]. We performed metabolite set enrichment analysis using Metaboanalyst 4.0 (RRID:SCR_015539) [13] for the extracted metabolites.

Gene expression analysis of treated cells

We seeded neuroblastoma cells (IMR-32 and SK-N-SH) and treated them with recombinant arginine deiminase and CBR-5884 in the same manner as that followed in metabolome analysis. We extracted the intracellular RNA from treated cells using NucleoSpin RNA (MACHEREY-NAGEL) after 24 h of CBR-5884 administration. The procedures for library preparation for RNA sequence, next-generation sequencing, alignment, quality check, and normalization of gene expression counts were the same as those used for the UT cohort tumor samples.

Mouse models

IMR-32 or SK-N-SH cells (both 5 × 10^6^ cells) in 50% Matrigel (Corning) and 50% RPMI were subcutaneously injected into the flanks of NOD/Shi-scid, IL-2RγKO (NOG) mice aged 6–8 weeks (CLEA Japan, Inc.). We used the littermates as controls in all experiments. Tumor volume was calculated as length × (width)^2^ / 2. We used NCT-503 (Cayman) for PHGDH inhibition and ADI-PEG20 for arginine depletion because of their stability *in vivo* [10, 14]. We randomized the mice and initiated the following treatments when the tumor size reached 100 mm^3^: for the NCT-503 experiments, mice were treated with NCT-503 (40 mg/kg daily, intraperitoneally) or a vehicle (3% DMSO, 35% PEG300 (both Tokyo Chemical Industries) and 62% of aqueous 30% hydroxypropyl-β-cyclodextrin (Sigma) [14]; and for ADI-PEG20 experiments, mice were treated with ADI-PEG20 (15 mg/kg intraperitoneally, once or twice a week) or a vehicle (saline) as reported previously in other tumors [15]. Five mice were treated in each group. Blinding was not used. Mice were treated for maximally 17 days and euthanized when the tumor size reached 2 000 mm^3^.

Statistical analyses

All statistical analyses were conducted using the R v3.5.3 software [16]. We evaluated the statistical significance for functional assays using the Student’s two-tailed t-test or the Wilcoxon rank-sum test, and a *P* value of <0.05 was considered statistically significant. We calculated the combination index using CompuSyn to evaluate the synergistic effect of multiple drugs [17].

Ethics approval involving animal studies

All procedures involving animal studies complied with the animal experimentation regulations of the University of Tokyo based on the International Guiding Principles for Biomedical Research Involving Animals. The Research Ethics Committee of the University of Tokyo approved all study protocols (approval number: 1598).

**Data availability**

RNA-seq and DNA methylation array data obtained in the current study were deposited in the DNA Data Bank of Japan (DDBJ) under the accession number hum0035 (JGAS000246 and JGAS000453).

**References**

1 Kimura S, Sekiguchi M, Watanabe K, Hiwatarai M, Seki M, Yoshida K *et al*. Association of high-risk neuroblastoma classification based on expression profiles with differentiation and metabolism. *PLoS One* 2021; 16: e0245526.

2 Pugh TJ, Morozova O, Attiyeh EF, Asgharzadeh S, Wei JS, Auclair D *et al*. The genetic landscape of high-risk neuroblastoma. *Nat Genet* (10.1038/ng.2529) 2013; 45: 279-284.

3 Asgharzadeh S, Pique-Regi R, Sposto R, Wang H, Yang Y, Shimada H *et al*. Prognostic significance of gene expression profiles of metastatic neuroblastomas lacking MYCN gene amplification. *J Natl Cancer Inst* 2006; 98: 1193-1203.

4 Barrett T, Wilhite SE, Ledoux P, Evangelista C, Kim IF, Tomashevsky M *et al*. NCBI GEO: archive for functional genomics data sets--update. *Nucleic Acids Res* 2013; 41: D991-995.

5 Morris TJ, Butcher LM, Feber A, Teschendorff AE, Chakravarthy AR, Wojdacz TK *et al*. ChAMP: 450k Chip Analysis Methylation Pipeline. *Bioinformatics* 2014; 30: 428-430.

6 Wilkerson MD, Hayes DN. ConsensusClusterPlus: a class discovery tool with confidence assessments and item tracking. *Bioinformatics* 2010; 26: 1572-1573.

7 Anders S, Huber W. Differential expression analysis for sequence count data. *Genome Biol* 2010; 11: R106.

8 McManus MT, Sharp PA. Gene silencing in mammals by small interfering RNAs. *Nat Rev Genet* 2002; 3: 737-747.

9 Ngo B, Kim E, Osorio-Vasquez V, Doll S, Bustraan S, Liang RJ *et al*. Limited Environmental Serine and Glycine Confer Brain Metastasis Sensitivity to PHGDH Inhibition. *Cancer Discov* 2020; 10: 1352-1373.

10 Feun L, Savaraj N. Pegylated arginine deiminase: a novel anticancer enzyme agent. *Expert Opin Investig Drugs* 2006; 15: 815-822.

11 Mullarky E, Lucki NC, Beheshti Zavareh R, Anglin JL, Gomes AP, Nicolay BN *et al*. Identification of a small molecule inhibitor of 3-phosphoglycerate dehydrogenase to target serine biosynthesis in cancers. *Proc Natl Acad Sci U S A* 2016; 113: 1778-1783.

12 Junker BH, Klukas C, Schreiber F. VANTED: a system for advanced data analysis and visualization in the context of biological networks. *BMC Bioinformatics* 2006; 7: 109.

13 Chong J, Soufan O, Li C, Caraus I, Li S, Bourque G *et al*. MetaboAnalyst 4.0: towards more transparent and integrative metabolomics analysis. *Nucleic Acids Res* 2018; 46: W486-W494.

14 Pacold ME, Brimacombe KR, Chan SH, Rohde JM, Lewis CA, Swier LJ *et al*. A PHGDH inhibitor reveals coordination of serine synthesis and one-carbon unit fate. *Nat Chem Biol* 2016; 12: 452-458.

15 Ji JX, Cochrane DR, Tessier-Cloutier B, Chen SY, Ho G, Pathak KV *et al*. Arginine Depletion Therapy with ADI-PEG20 Limits Tumor Growth in Argininosuccinate Synthase-Deficient Ovarian Cancer, Including Small-Cell Carcinoma of the Ovary, Hypercalcemic Type. *Clin Cancer Res* 2020; 26: 4402-4413.

16 R Core Team. R: A language and environment for statistical computing. R Foundation for Statistical Computing: Vienna, Austria., 2017.

17 Chou TC, Talalay P. Quantitative analysis of dose-effect relationships: the combined effects of multiple drugs or enzyme inhibitors. *Adv Enzyme Regul* 1984; 22: 27-55.
