# Supplementary figures and images for: Identification of the ultrahigh-risk subgroup in neuroblastoma cases through DNA methylation analysis and its treatment exploiting cancer metabolism
Source: Oncogene. 2022 Nov 1;41(46):4994–5007. doi: 10.1038/s41388-022-02489-2 (PMC9652143; doi:10.1038/s41388-022-02489-2)

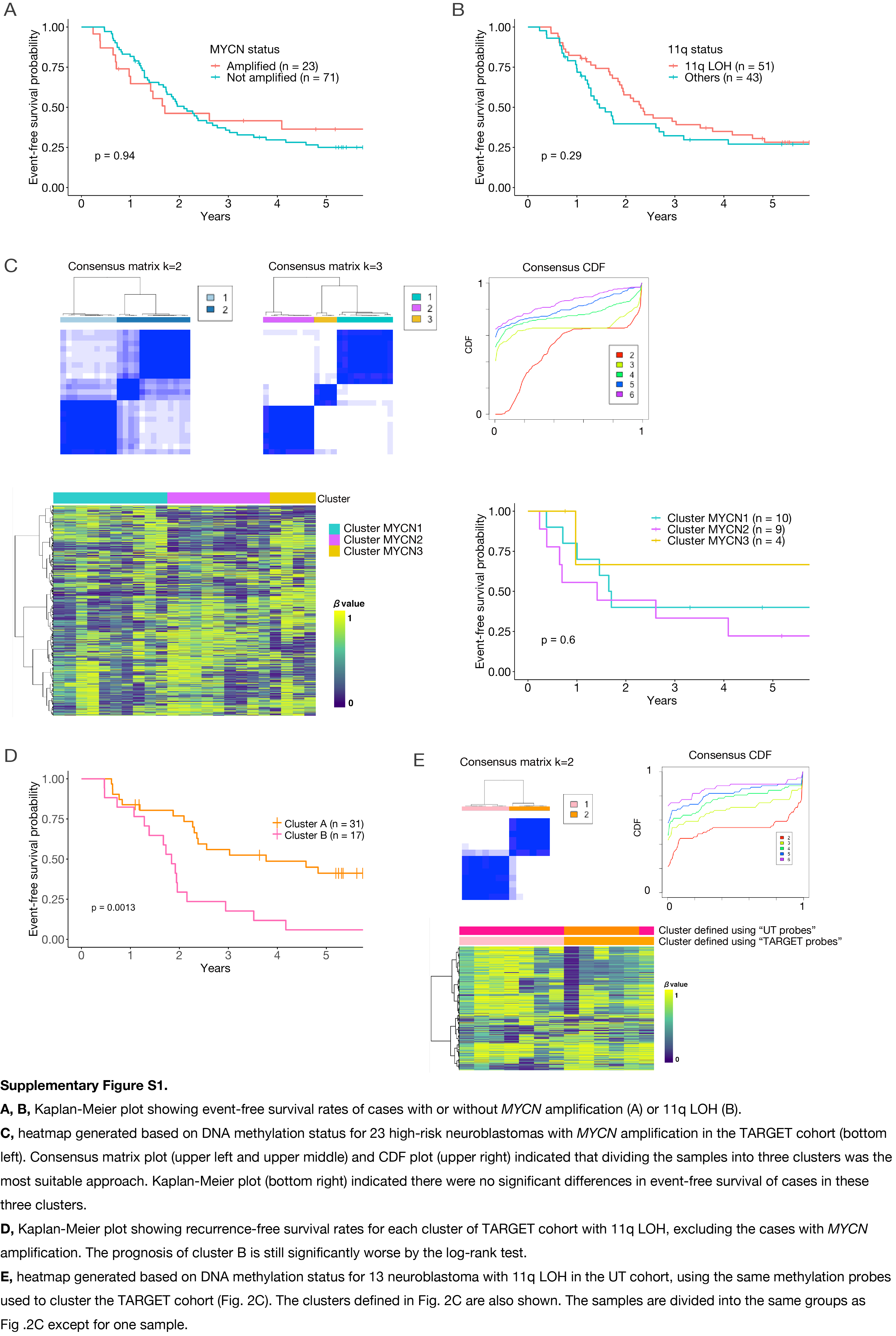

Supplement: Supplementary file 2 — Supplementary Figure S1 [file 41388_2022_2489_MOESM2_ESM.png]

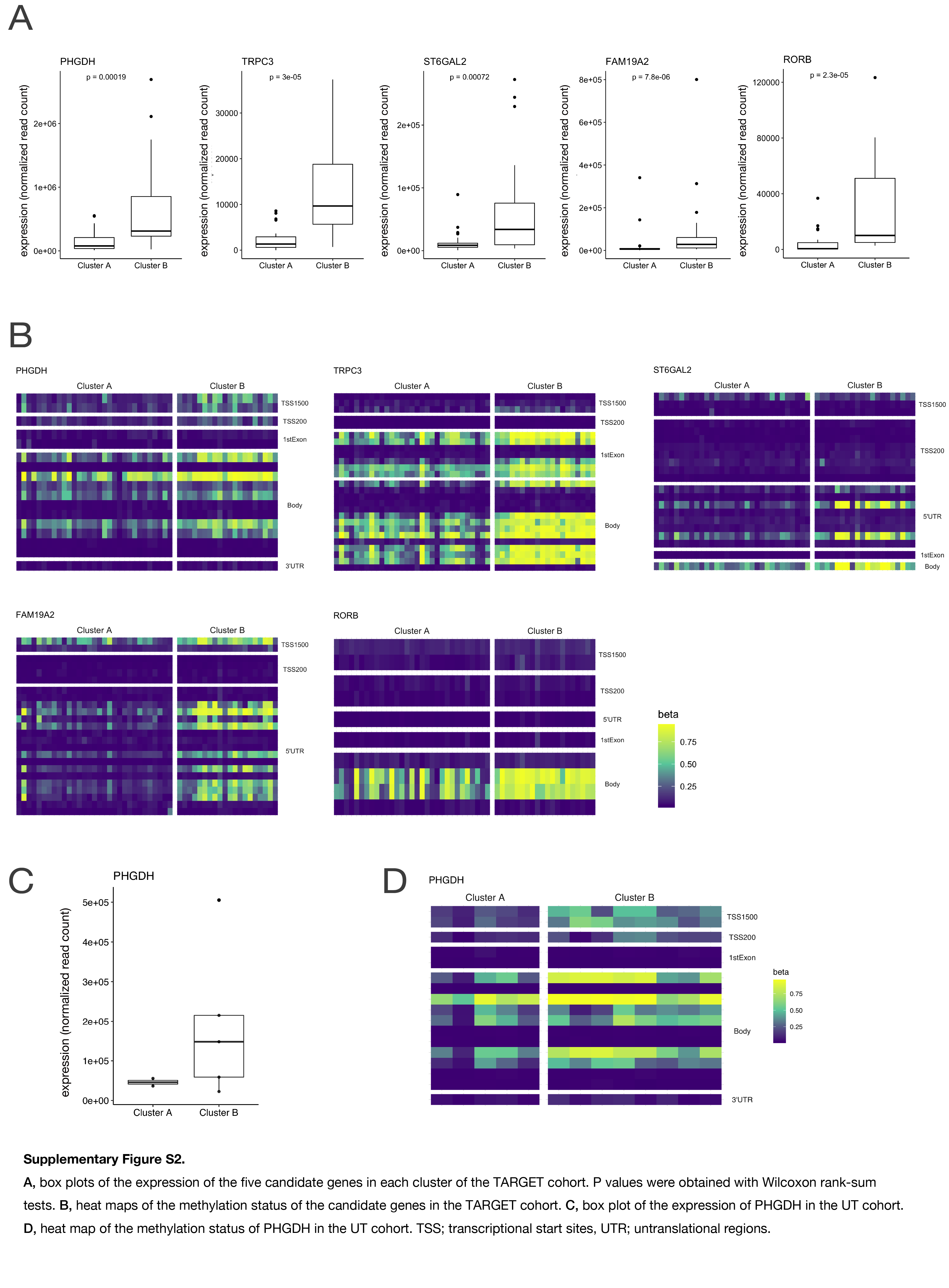

Supplement: Supplementary file 3 — Supplementary Figure S2 [file 41388_2022_2489_MOESM3_ESM.png]

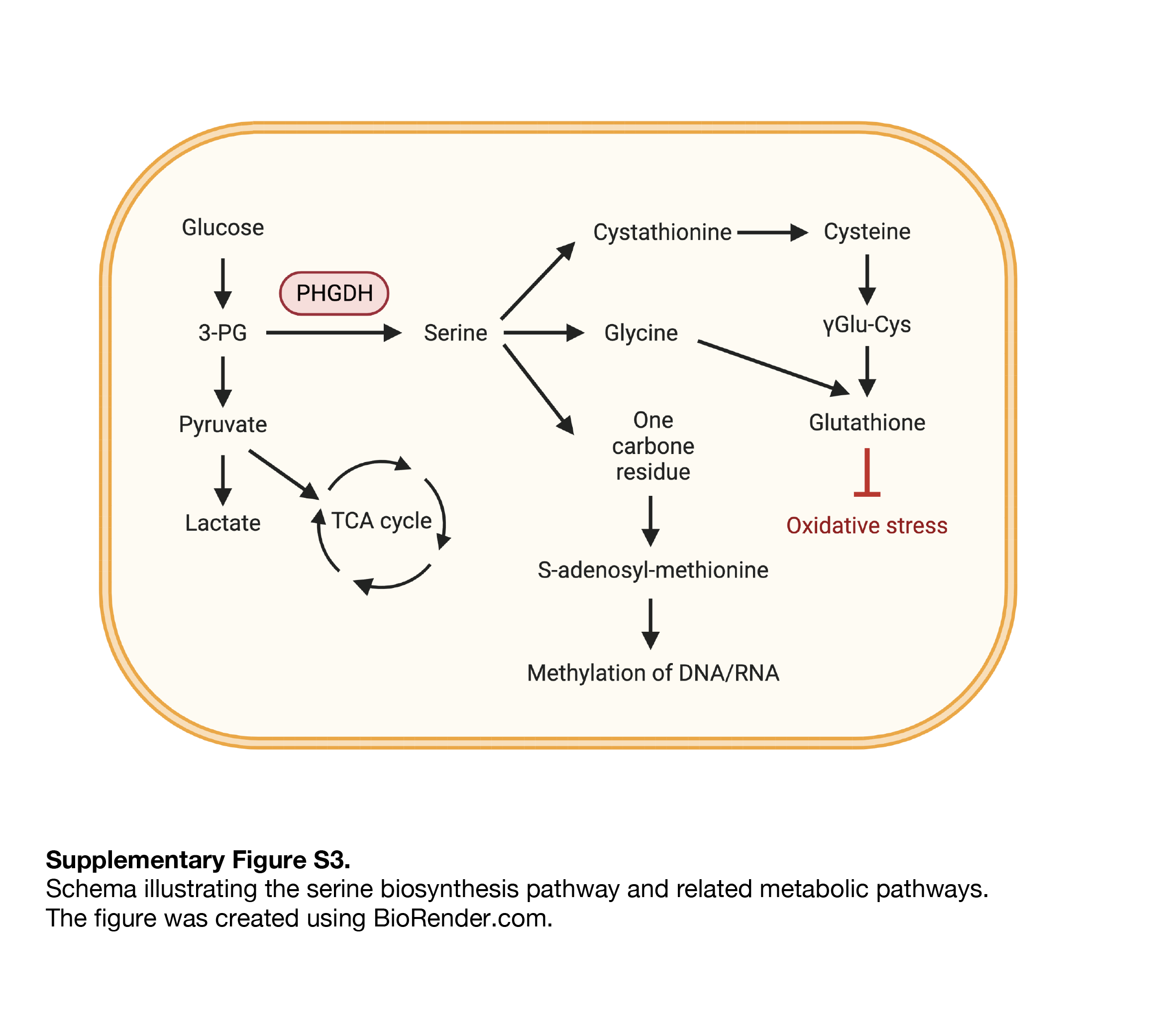

Supplement: Supplementary file 4 — Supplementary Figure S3 [file 41388_2022_2489_MOESM4_ESM.png]

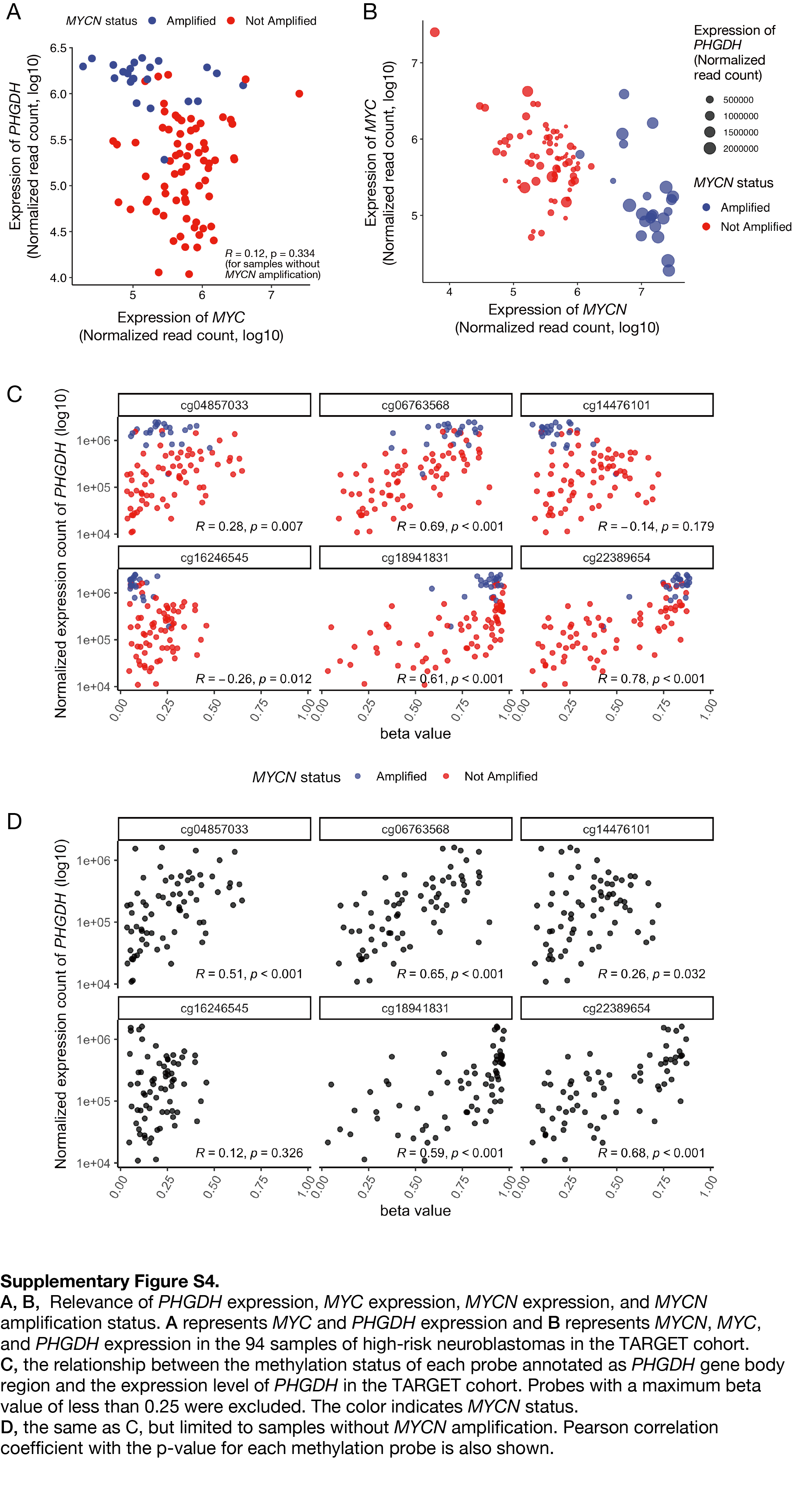

Supplement: Supplementary file 5 — Supplementary Figure S4 [file 41388_2022_2489_MOESM5_ESM.png]

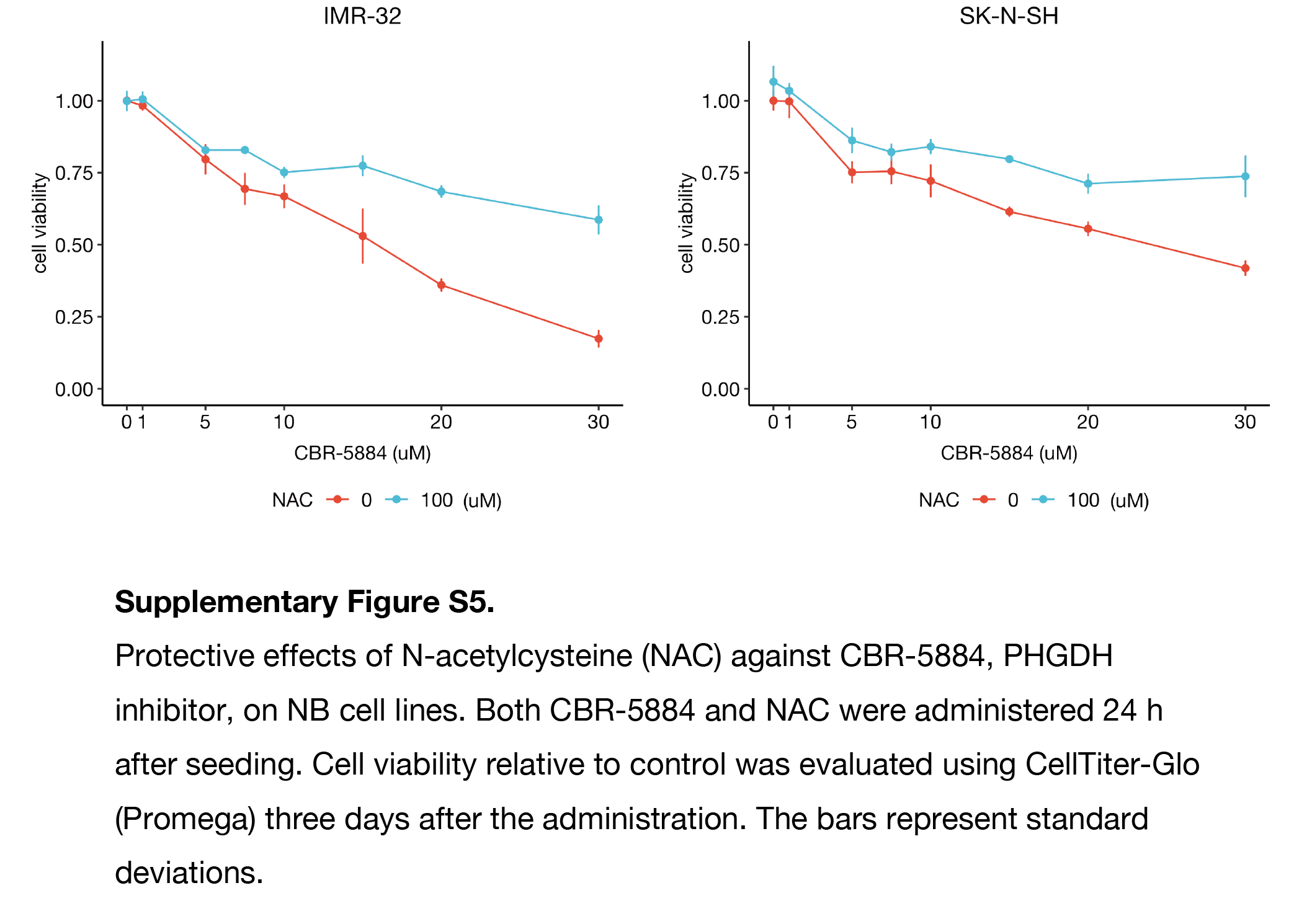

Supplement: Supplementary file 6 — Supplementary Figure S5 [file 41388_2022_2489_MOESM6_ESM.png]

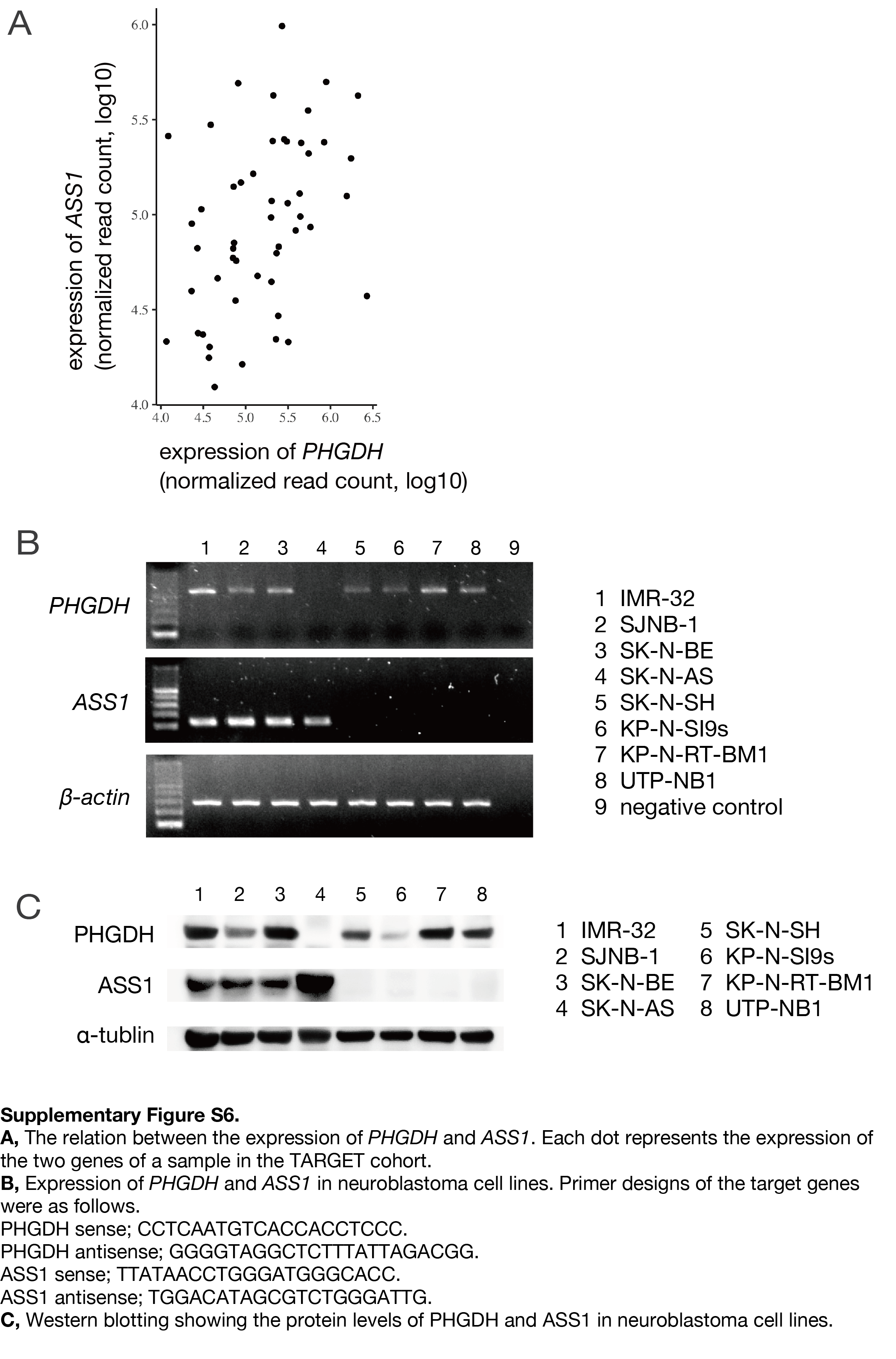

Supplement: Supplementary file 7 — Supplementary Figure S6 [file 41388_2022_2489_MOESM7_ESM.png]

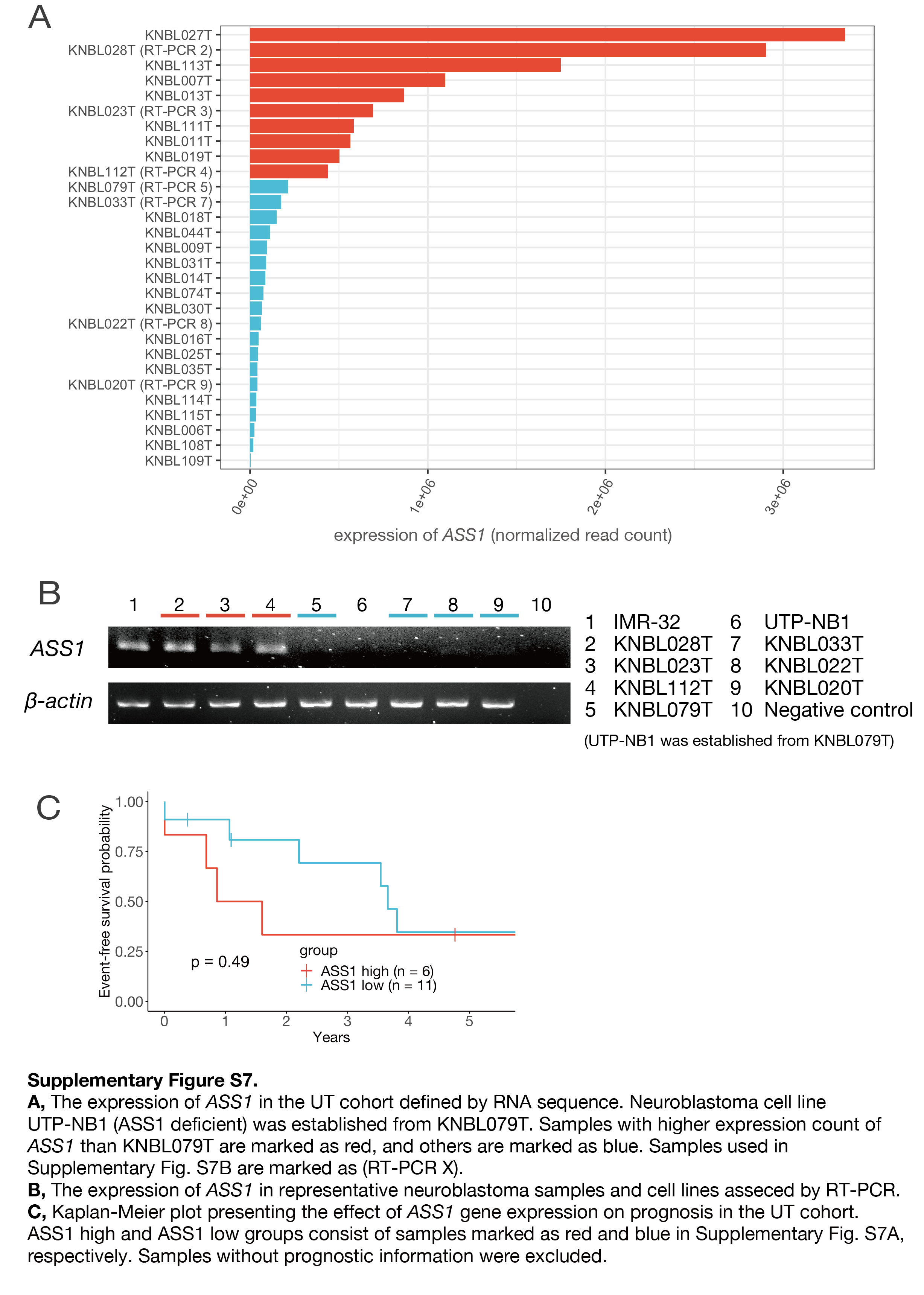

Supplement: Supplementary file 8 — Supplementary Figure S7 [file 41388_2022_2489_MOESM8_ESM.png]

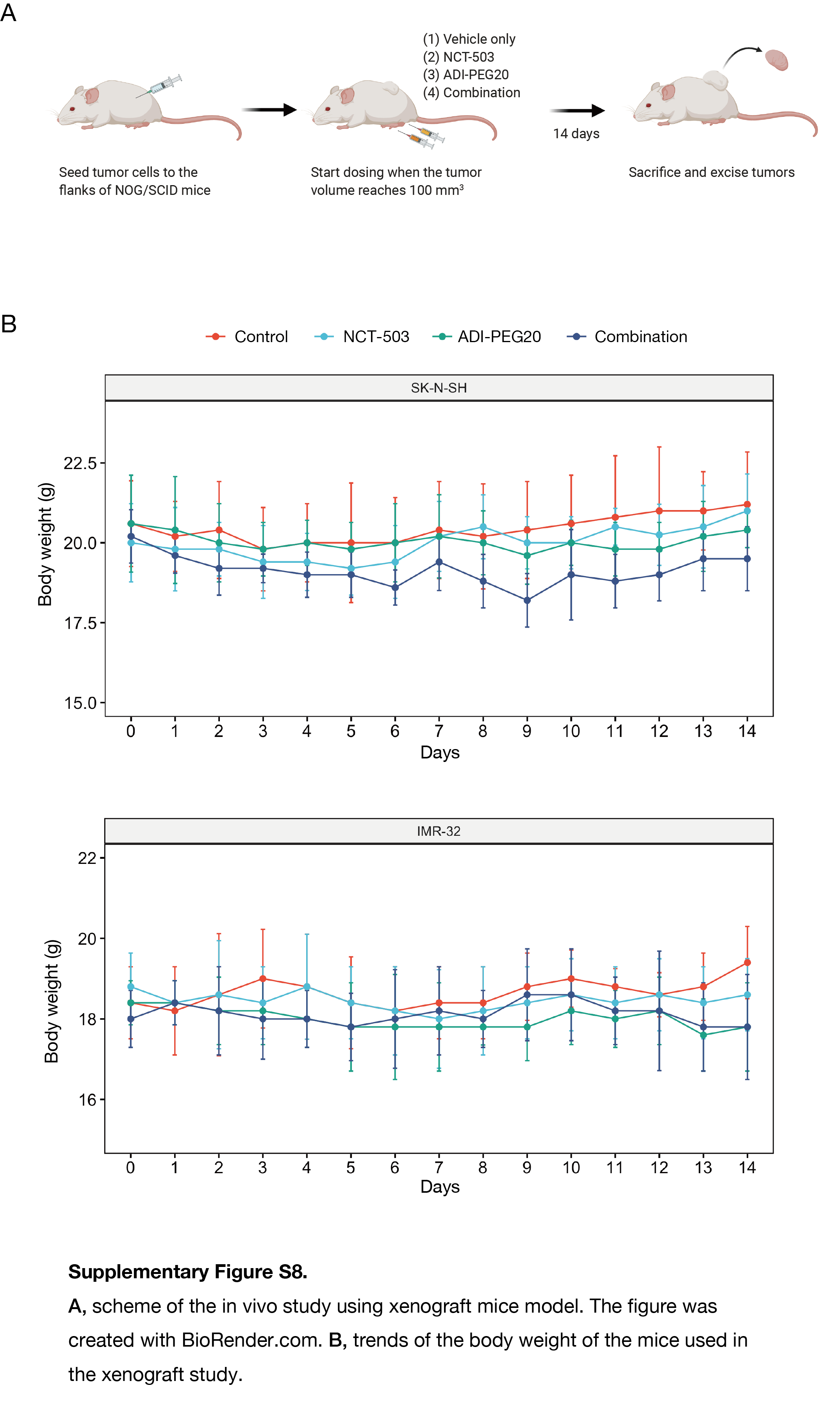

Supplement: Supplementary file 9 — Supplementary Figure S8 [file 41388_2022_2489_MOESM9_ESM.png]

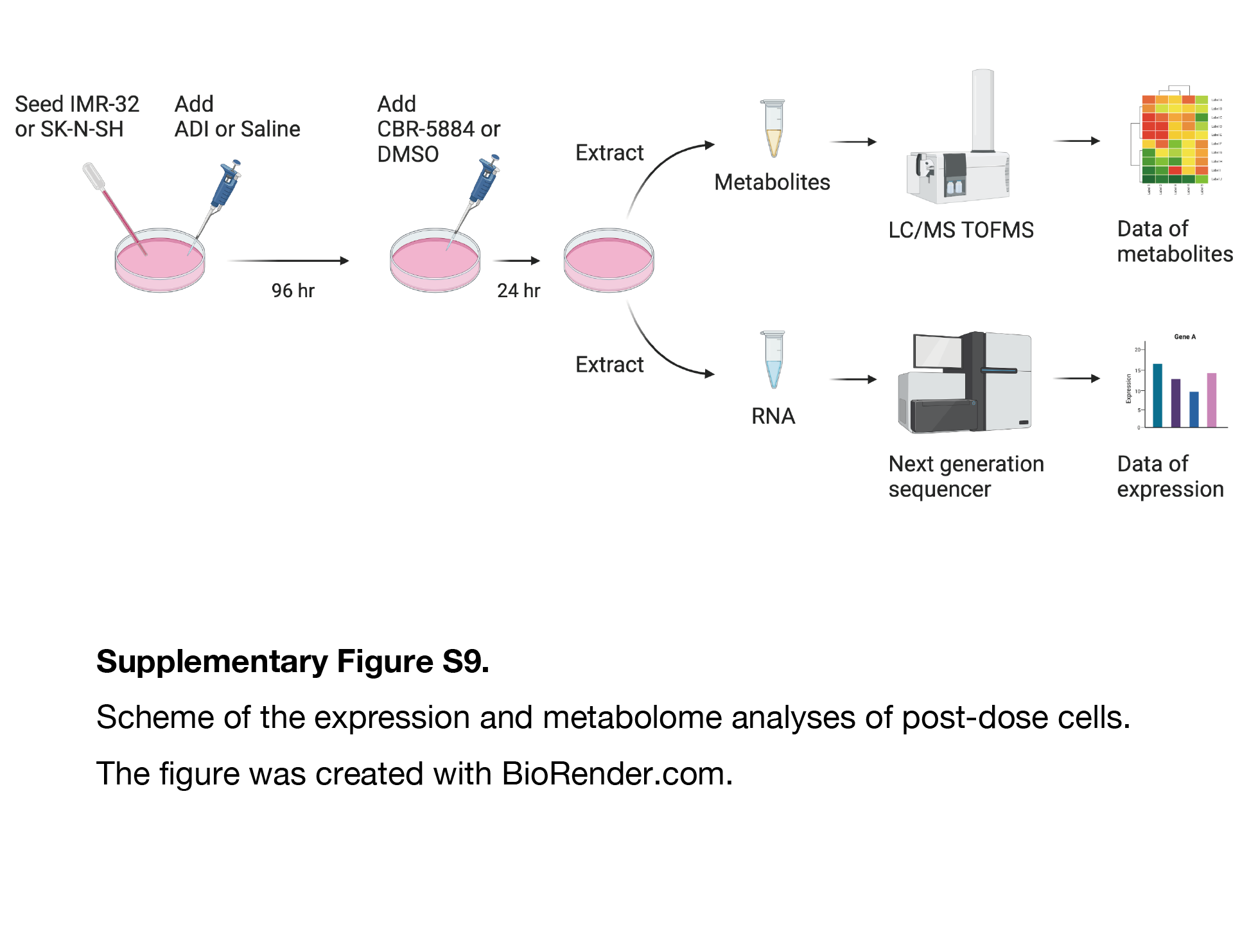

Supplement: Supplementary file 10 — Supplementary Figure S9 [file 41388_2022_2489_MOESM10_ESM.png]

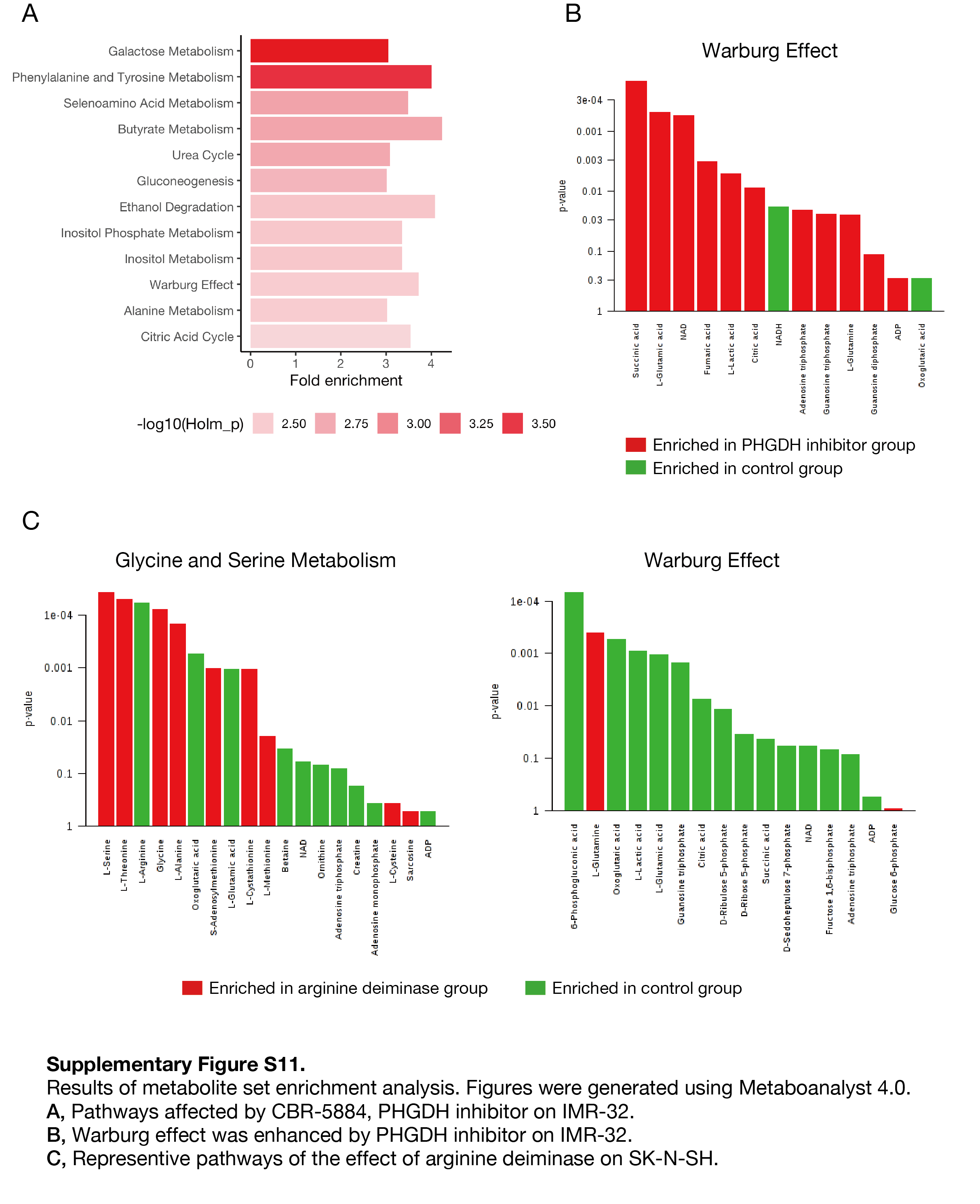

Supplement: Supplementary file 12 — Supplementary Figure S11 [file 41388_2022_2489_MOESM12_ESM.png]
