# Supplementary material for: Identification of the ultrahigh-risk subgroup in neuroblastoma cases through DNA methylation analysis and its treatment exploiting cancer metabolism
Source: Oncogene. 2022 Nov 1;41(46):4994–5007. doi: 10.1038/s41388-022-02489-2 (PMC9652143; doi:10.1038/s41388-022-02489-2)
Supplement: Supplementary file 11 — Supplementary Figure S10 [file 41388_2022_2489_MOESM11_ESM.pdf]

A

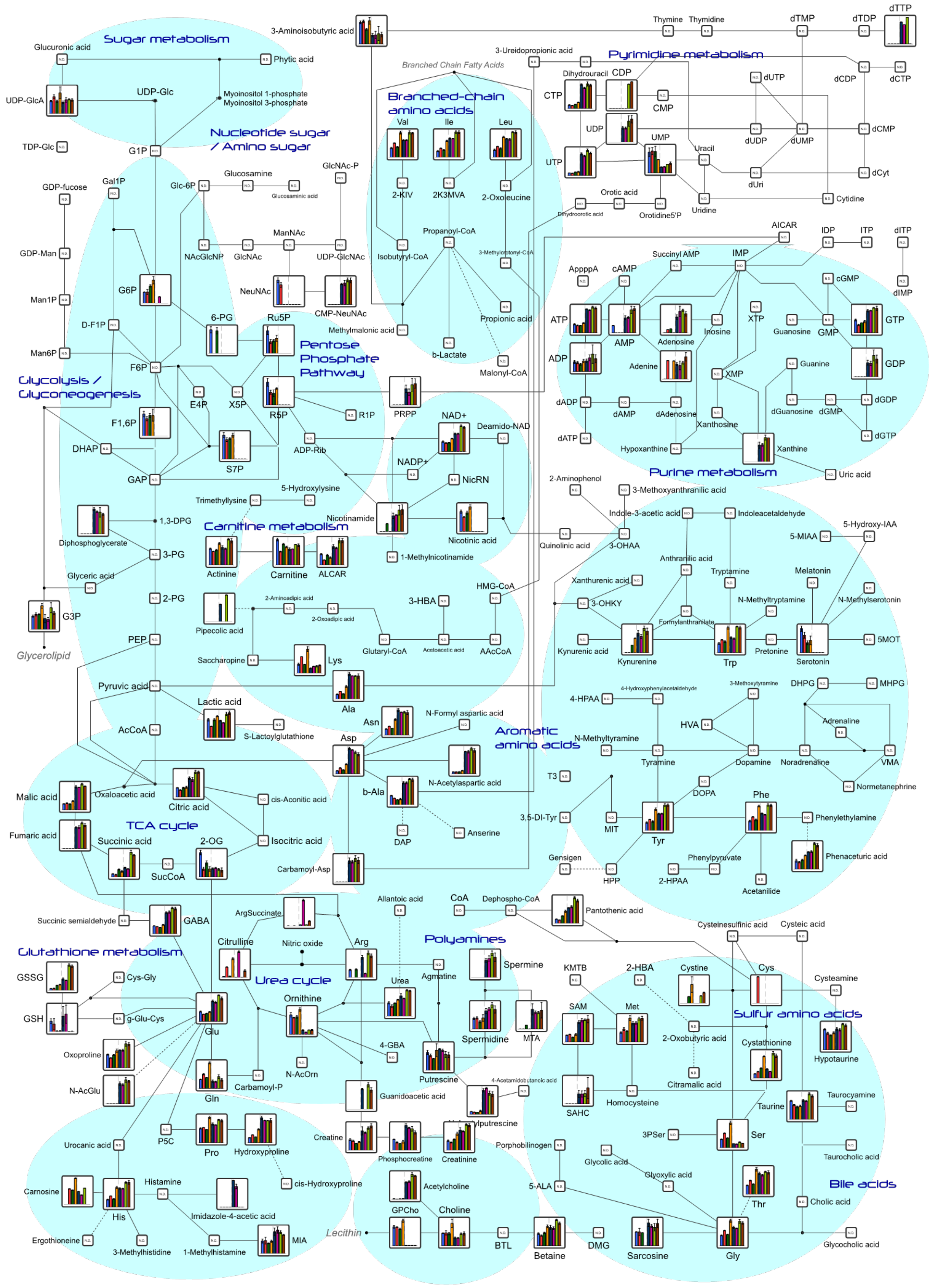

Supplementary Figure S10.

Metabolic pathway maps representing the relative amounts of the detected metabolites. Bar charts indicate SK-N-SH control (blue), SK-N-SH with arginine deiminase (red), SK-N-SH with PHGDH inhibitor (green), SK-N-SH with both arginine deiminase and PHGDH inhibitor (orange), IMR-32 control (navy), IMR-32 with arginine deiminase (purple), IMR-32 with PHGDH inhibitor (light green), and IMR-32 with both arginine deiminase and PHGDH inhibitor (brown), respectively. **A,** the overall map. **B,** central carbon metabolism. **C,** urea cycle and its related pathways. **D,** lipid metabolism and its related pathways. **E,** branched-chain amino acid and aromatic amino acid metabolism. **F,** purine and pyrimidine metabolism. **G,** coenzyme metabolism.

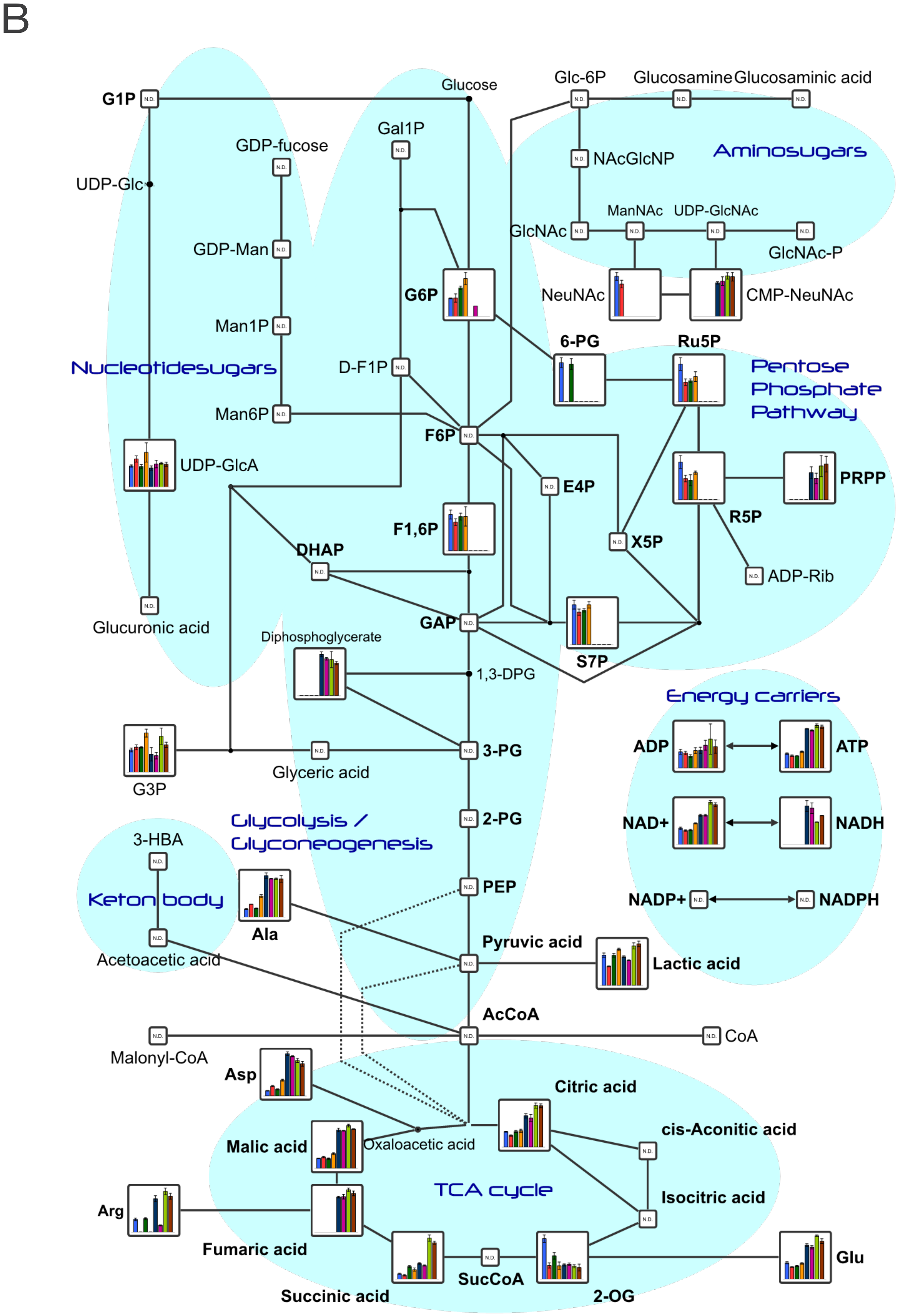
B
G1P
Glucose
Glc-6P
Glucosamine
Glucosaminic acid
Gal1P
GDP-fucose
UDP-Glc
NAcGlcNP
Aminosugars
ManNAc
UDP-GlcNAc
GlcNAc
GDP-Man
GlcNAc-P
G6P
NeuNAc
CMP-NeuNAc
Man1P
6-PG
Ru5P
Nucleotidesugars
D-F1P
Pentose Phosphate Pathway
Man6P
F6P
UDP-GlcA
E4P
PRPP
R5P
F1,6P
DHAP
X5P
ADP-Rib
Glucuronic acid
GAP
Diphosphoglycerate
S7P
1,3-DPG
Energy carriers
ADP
ATP
G3P
3-PG
Glyceric acid
NAD+
NADH
2-PG
Glycolysis / Glyconeogenesis
3-HBA
Keton body
NADP+
NADPH
PEP
Ala
Acetoacetic acid
Pyruvic acid
Lactic acid
AcCoA
Malonyl-CoA
CoA
Asp
Citric acid
cis-Aconitic acid
Malic acid
Oxaloacetic acid
TCA cycle
Isocitric acid
Arg
Fumaric acid
SucCoA
Glu
Succinic acid
2-OG
N.D.

C

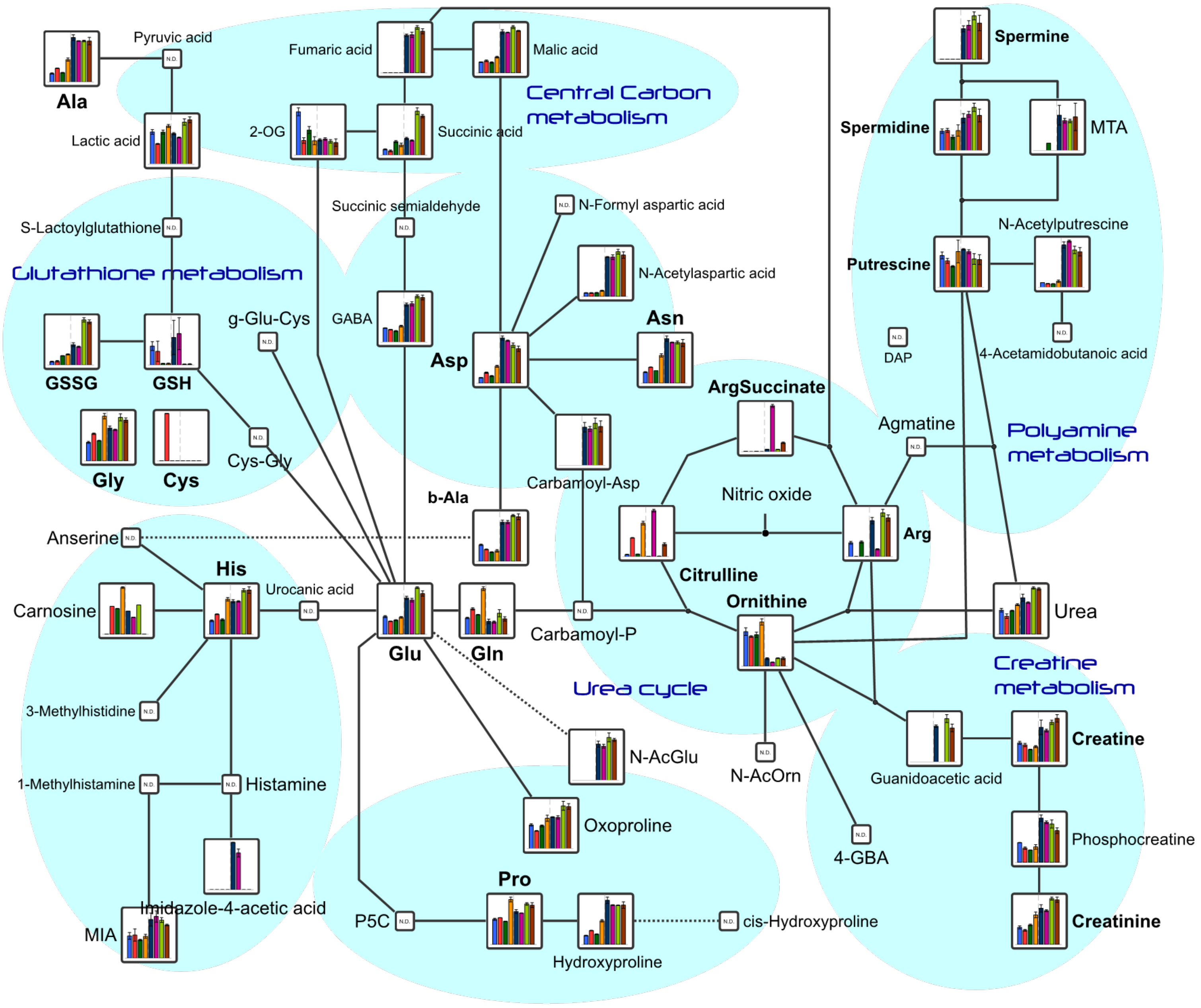

Pyruvic acid
Ala
Lactic acid
Fumaric acid
Malic acid
2-OG
Succinic acid
Central Carbon metabolism
S-Lactoylglutathione
Succinic semialdehyde
N-Formyl aspartic acid
Glutathione metabolism
N-Acetylaspartic acid
g-Glu-Cys
GABA
Asn
GSSG
GSH
Asp
Gly
Cys
Cys-Gly
ArgSuccinate
Carbamoyl-Asp
b-Ala
Nitric oxide
Anserine
His
Urocanic acid
Carnosine
Glu
Gln
Carbamoyl-P
Citrulline
Ornithine
Arg
Urea cycle
3-Methylhistidine
N-AcGlu
N-AcOrn
1-Methylhistamine
Histamine
Oxoproline
Imidazole-4-acetic acid
MIA
Pro
P5C
Hydroxyproline
cis-Hydroxyproline
Spermine
Spermidine
MTA
N-Acetylputrescine
Putrescine
DAP
4-Acetamidobutanoic acid
Agmatine
Polyamine metabolism
Urea
Creatine metabolism
Guanidoacetic acid
Creatine
4-GBA
Phosphocreatine
Creatinine
N.D.

D

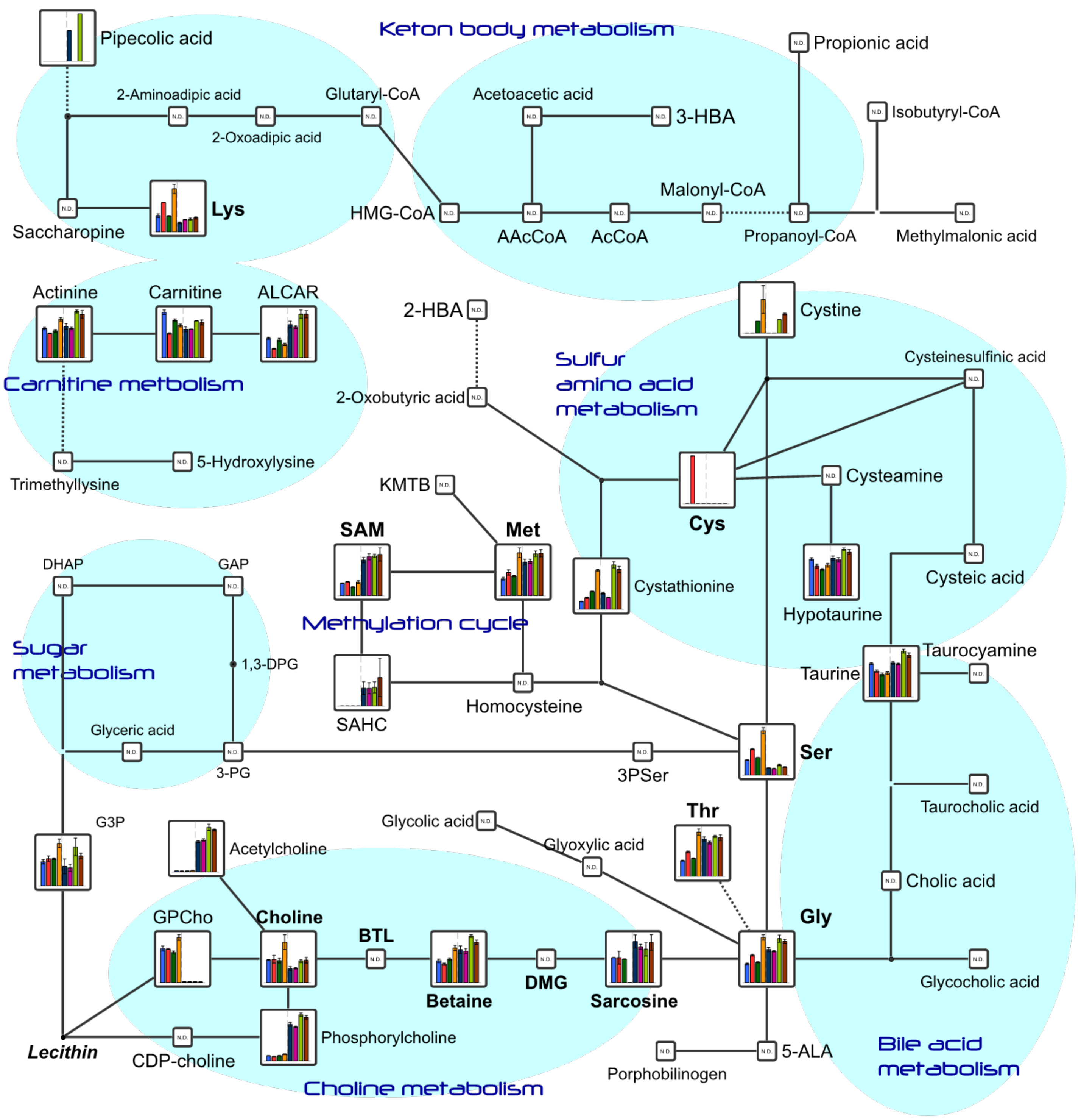
Keton body metabolism
Pipecolic acid
Propionic acid
2-Aminoadipic acid
Glutaryl-CoA
Acetoacetic acid
3-HBA
Isobutyryl-CoA
2-Oxoadipic acid
Malonyl-CoA
Lys
HMG-CoA
Saccharopine
AAcCoA
AcCoA
Propanoyl-CoA
Methylmalonic acid
Actinine
Carnitine
ALCAR
2-HBA
Cystine
Sulfur amino acid metabolism
Cysteinesulfinic acid
Carnitine metbolism
2-Oxobutyric acid
Trimethyllysine
5-Hydroxylysine
Cysteamine
KMTB
Cys
SAM
Met
DHAP
GAP
Cystathionine
Hypotaurine
Cysteic acid
Methylation cycle
Sugar metabolism
1,3-DPG
Taurocyamine
Taurine
SAHC
Homocysteine
Glyceric acid
Ser
3-PG
3PSer
Taurocholic acid
Thr
G3P
Glycolic acid
Acetylcholine
Glyoxylic acid
Cholic acid
GPCho
Choline
BTL
Gly
Betaine
DMG
Sarcosine
Glycocholic acid
Lecithin
CDP-choline
Phosphorylcholine
5-ALA
Porphobilinogen
Bile acid metabolism
Choline metabolism
N.D.

E

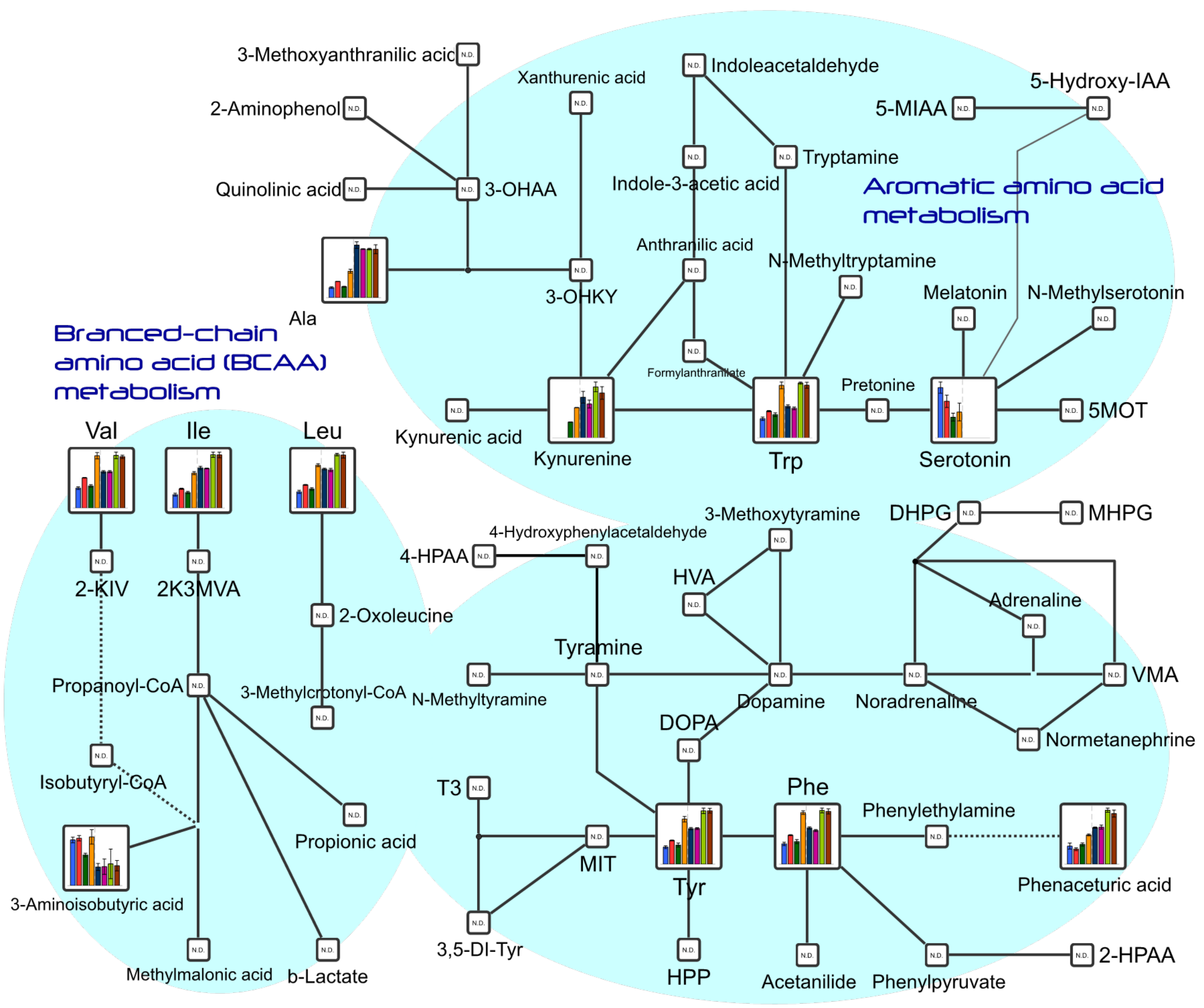

3-Methoxyanthranilic acid
2-Aminophenol
Quinolinic acid
3-OHAA
Xanthurenic acid
Indoleacetaldehyde
Tryptamine
Indole-3-acetic acid
5-Hydroxy-IAA
5-MIAA
Aromatic amino acid metabolism
Anthranilic acid
N-Methyltryptamine
Melatonin
N-Methylserotonin
Ala
3-OHKY
Formylanthranilate
Pretonine
Branced-chain amino acid (BCAA) metabolism
Kynurenic acid
Kynurenine
Trp
Serotonin
5MOT
Val
Ile
Leu
3-Methoxytyramine
DHPG
MHPG
4-Hydroxyphenylacetaldehyde
4-HPAA
2-KIV
2K3MVA
HVA
2-Oxoleucine
Adrenaline
Tyramine
Propanoyl-CoA
3-Methylcrotonyl-CoA
N-Methyltyramine
Dopamine
Noradrenaline
VMA
Normetanephrine
DOPA
Isobutyryl-CoA
T3
Phe
Phenylethylamine
Propionic acid
MIT
Tyr
Phenaceturic acid
3-Aminoisobutyric acid
3,5-DI-Tyr
HPP
Acetanilide
Phenylpyruvate
2-HPAA
Methylmalonic acid
b-Lactate
N.D.

F

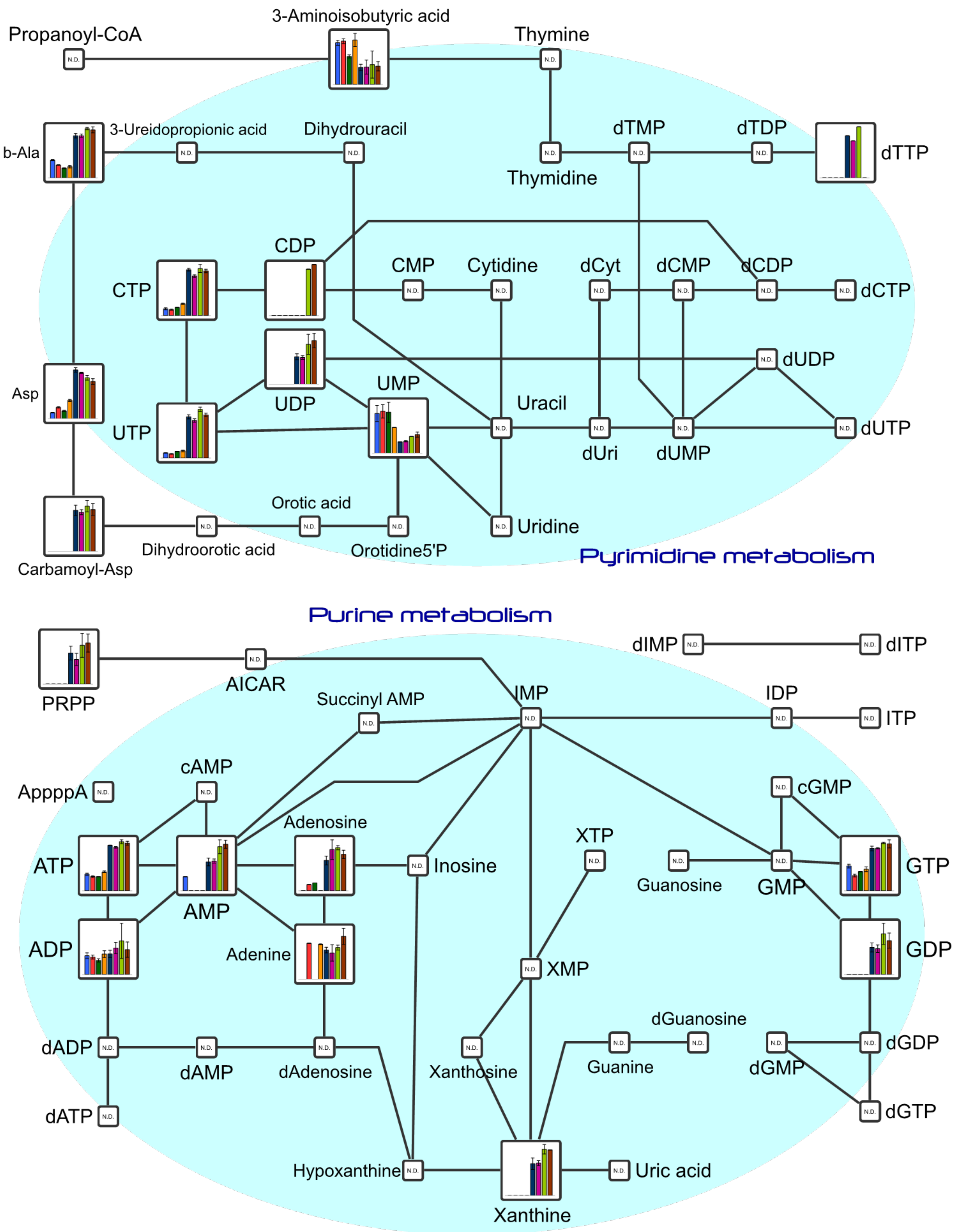

Propanoyl-CoA
3-Aminoisobutyric acid
Thymine
b-Ala
3-Ureidopropionic acid
Dihydrouracil
dTMP
dTDP
dTTP
Thymidine
CDP
CTP
CMP
Cytidine
dCyt
dCMP
dCDP
dCTP
dUDP
UDP
UMP
Asp
Uracil
UTP
dUri
dUMP
dUTP
Orotic acid
Uridine
Dihydroorotic acid
Orotidine5'P
Carbamoyl-Asp
Pyrimidine metabolism
Purine metabolism
dIMP
dITP
AICAR
PRPP
IMP
IDP
ITP
Succinyl AMP
cAMP
ApppppA
cGMP
Adenosine
XTP
ATP
Inosine
Guanosine
GMP
GTP
AMP
ADP
Adenine
XMP
GDP
dGuanosine
dADP
dAMP
dAdenosine
Xanthosine
Guanine
dGMP
dGDP
dATP
dGTP
Hypoxanthine
Uric acid
Xanthine
N.D.

G

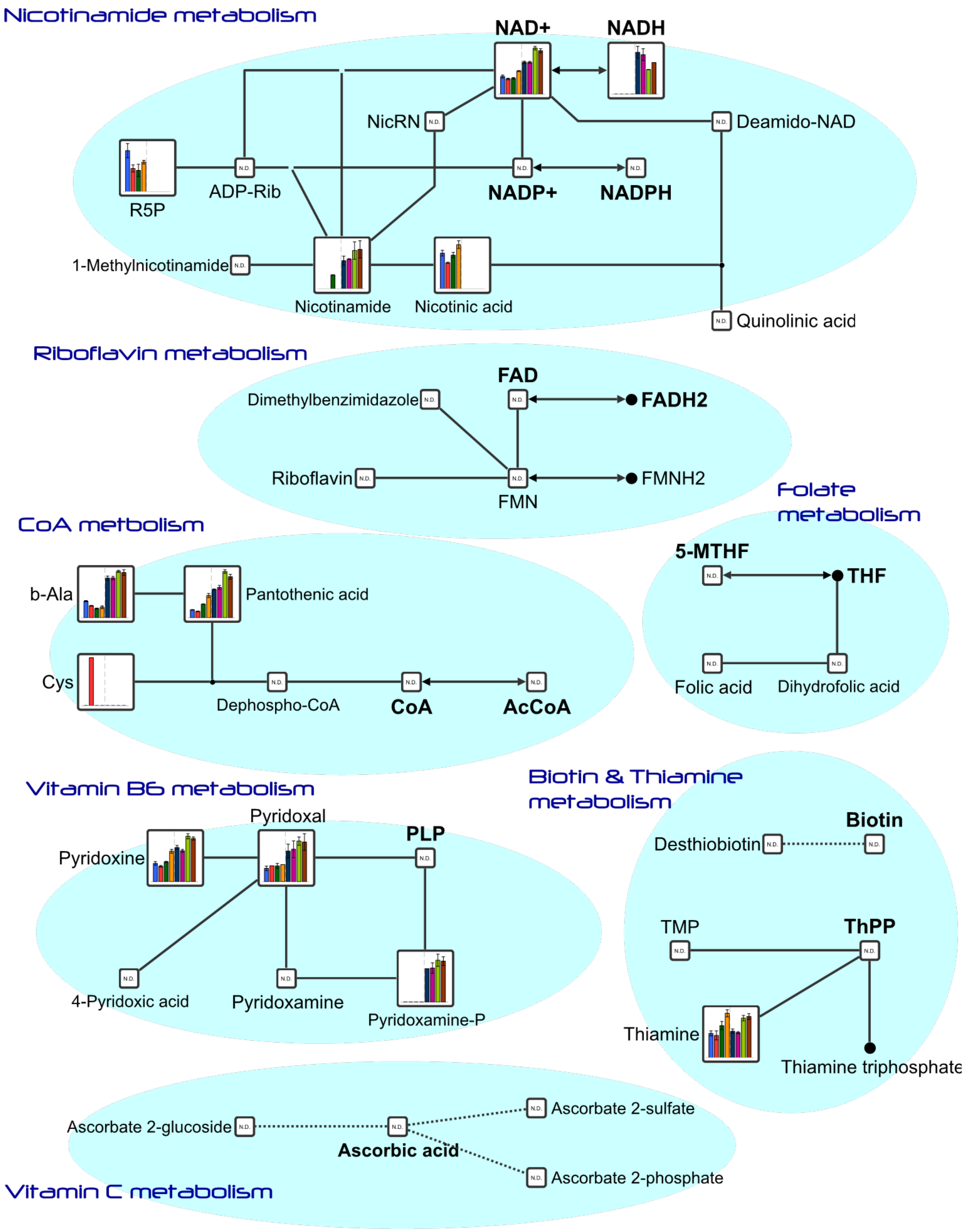

Nicotinamide metabolism
NAD+
NADH
NicRN
Deamido-NAD
R5P
ADP-Rib
NADP+
NADPH
1-Methylnicotinamide
Nicotinamide
Nicotinic acid
Quinolinic acid
Riboflavin metabolism
FAD
Dimethylbenzimidazole
FADH2
Riboflavin
FMN
FMNH2
Folate metabolism
CoA metbolism
5-MTHF
THF
b-Ala
Pantothenic acid
Cys
Dephospho-CoA
CoA
AcCoA
Folic acid
Dihydrofolic acid
Vitamin B6 metabolism
Biotin & Thiamine metabolism
Pyridoxal
PLP
Pyridoxine
Biotin
Desthiobiotin
4-Pyridoxic acid
Pyridoxamine
Pyridoxamine-P
TMP
ThPP
Thiamine
Thiamine triphosphate
Ascorbate 2-glucoside
Ascorbic acid
Ascorbate 2-sulfate
Ascorbate 2-phosphate
Vitamin C metabolism
N.D.
